# Topological comparison of methods for predicting transcriptional cooperativity in yeast

**DOI:** 10.1186/1471-2164-9-137

**Published:** 2008-03-25

**Authors:** Daniel Aguilar, Baldo Oliva

**Affiliations:** 1 Structural Bioinformatics Group (GRIB), IMIM-Universitat Pompeu Fabra, C/Doctor Aiguader, 88, Barcelona 08003, Spain

## Abstract

**Background:**

The cooperative interaction between transcription factors has a decisive role in the control of the fate of the eukaryotic cell. Computational approaches for characterizing cooperative transcription factors in yeast, however, are based on different rationales and provide a low overlap between their results. Because the wealth of information contained in protein interaction networks and regulatory networks has proven highly effective in elucidating functional relationships between proteins, we compared different sets of cooperative transcription factor pairs (predicted by four different computational methods) within the frame of those networks.

**Results:**

Our results show that the overlap between the sets of cooperative transcription factors predicted by the different methods is low yet significant. Cooperative transcription factors predicted by all methods are closer and more clustered in the protein interaction network than expected by chance. On the other hand, members of a cooperative transcription factor pair neither seemed to regulate each other nor shared similar regulatory inputs, although they do regulate similar groups of target genes.

**Conclusion:**

Despite the different definitions of transcriptional cooperativity and the different computational approaches used to characterize cooperativity between transcription factors, the analysis of their roles in the framework of the protein interaction network and the regulatory network indicates a common denominator for the predictions under study. The knowledge of the shared topological properties of cooperative transcription factor pairs in both networks can be useful not only for designing better prediction methods but also for better understanding the complexities of transcriptional control in eukaryotes.

## Background

Current studies indicate that the combinatorial control of transcription allows an extremely large number of regulatory decisions (particularly in eukaryotes) through the cooperation of a small number of transcription factors (TFs) [1-3]. Determining cooperativity between TFs is essential to understand transcriptional regulation. However, in contrast to other well-characterized relationships between proteins, cooperativity in a broad sense does not have a unique description. It has been simply described as the regulation of the expression of a gene by two or more specific transcription factors [4], often related to protein-protein interactions between the DNA-binding elements [5-8]. In this line, cooperation between TFs has been restricted to the existence of DNA-binding sites close in the same promoter regions of target genes [9]. However, other studies have suggested a basis for cooperativity in the role of cis-regulatory elements acting as analogue implementations of logic circuits, devoid of protein-protein contacts [10,11]. In addition, some works showed that cooperative TF pairs (hereinafter CTFPs) do not act necessarily together, neither spatially nor temporally [11-13]. A model by Cokus et al. assumed that all TFs binding the same promoter do cooperate with one another in some degree [14]. Finally, transcriptional synergy (a non-linear regulatory effect on the expression of a gene when two or more TFs bind its promoter) has also been considered as a form of cooperativity [15,16].

We investigated the nature of four sets of CTFPs (predicted by four different computational methods, see Table 1 and *Methods*) by means of the analysis of their roles in two distinct biological networks (the protein interaction network and the regulatory network). Our findings suggest that cooperativity is reflected in the structure of the protein interaction network (PIN) with shorter path lengths and larger topological overlaps (i.e. larger modularity) than expected by chance. This was true for all four sets of CTFPs, implying a common denominator in the nature of all the predictions regardless of the prediction method used. Also, members of CTFPs seem to share common target genes but do not show other distinctive regulatory traits, neither in terms of inter-regulation nor in terms of their in-degree (i.e. the regulatory influence upon them). Since cooperativity seems to be responsible for many important transcriptional responses in the cell, we believe that the results presented here will help to better understand its nature and, consequently, will assist in providing a solid framework to develop better tools for its prediction.

**Table 1:** Methods under study.

***Method***	***Rationale behind the method***	***CTFPs considered in our study***
**Method N **[20]	Proteins that are close in the PIN are likely to be co-regulated by the same TFs. Cooperative TF pairs are identified on the basis of the distance between their common target genes in the PIN (as opposed to the distance between genes controlled by either TF). Subcellular localization data was used to filter the PIN. Functional data was used to refine the distances between target genes.	Cooperative TF pairs, triads and modules. Members of triads and modules were pairwisely decomposed in an all-vs-all fashion. Gene names were transformed to YPD names. TFs not present in the set of 101 TFs common to all methods were excluded. The number of cooperative TF pairs was 45.
**Method B **[46]	Proteins with similar expression profiles are likely to be co-regulated. Cooperative TF pairs are identified on the basis of their influence on the cell-cycle-dependent co-expression of their common target genes.	Significant cooperative TF pairs labeled as significant (P_B _< 0.001). Gene names were transformed to YPD names and TFs not present in the set of 101 TFs common to all methods were excluded. The number of cooperative TF pairs was 31.
**Method T **[48]	Cooperativity has an influence in the expression level of regulated genes during one or more phases of the cell cycle. First, TFs involved in regulation of the cell cycle are found. Then, TF pairs associated to a target gene more than random expectation are identified. Of these, a cooperative interaction between two TFs is identified based on their influence in the expression level of the target genes regulated by them.	Only pairs labeled as "confident" considered. Gene names were transformed to YPD names and TFs not present in the set of 101 TFs common to all methods were excluded. The number of cooperative TF pairs was 15.
**Method C **[47]	DNA-binding sites of cooperative TFs are likely to co-occur in the target genes. Also, cooperative TF pairs are likely to influence changes in the expression profiles of target genes. This influence was measured by means of a dynamic stochastic model on cell-cycle expression data. The method was also applied to gene expression under H_2_O_2 _stress.	Only TF pairs with *p*-value < 10^-21 ^were considered. Gene names were transformed to YPD names and TFs not present in the set of 101 TFs common to all methods were excluded. The number of cooperative TF pairs was 46.

Features of the four methods under study. Abbreviations: TF, transcription factor; PIN, protein interaction network.

## Results and discussion

### Similarities and dependences between predictions

As no gold-standard exists for cooperative TF pairs, we compared the predictions of the four methods by means of their ability to predict the results of one another. We found that 32 (35.2%) of the CTFPs are predicted by more than one method and 8 (8.8%) are predicted by more than two. The fact that only 6 (6.6%) of the CTFPs are predicted by all four methods suggests that divergent criteria in characterizing cooperativity accounts for a large part of the observed divergence in the results of the four methods. In order to calculate the pairwise dependences and the overlap between the four datasets, we used the mutual information coefficient and the Jaccard coefficient, respectively [17-19]. Results are shown in Table 2. The predictions of the four methods are not significantly correlated to one another in terms of mutual information, although their overlap in terms of their positive predictions is low yet significant. The low level of this overlap also reveals largely divergent criteria to assess cooperativity. Indeed, as shown by the mutual information analysis, knowing the results of one method gives little information on the results expected in any other method. The different data sources used by each method might account for part of this observation. For example, the TF pair YLR131C (Ace2) – YGL073W (Hsf1) does not co-occur in the location data from Harbison et al. [9], so it could not be predicted by method T, which relied in this information source. However, it was characterized as cooperative by method B, which relied on a different data source. Also, the threshold values applied by each method affect the list of TF pairs accepted as cooperative. An additional explanation for the observed disagreements between results could be the criteria used to strengthen computational prediction of cooperativity by seeking support from experimental observations. Experimental support in the four papers considered in this study had different forms, for instance: (i) TF pairs which are known to physically interact (such as YER111C (Swi4) – YLR182W (Swi6), forming the SBF complex, or YDL056W (Mbp1) – YLR182W (Swi6), forming the MBF complex); (ii) TF pairs which belong to the same transcriptional complex (such as YOR372C (Ndd1) – YIL131C (Fhk1), which belong to the SFF complex despite the absence of recorded physical interaction between them); (iii) TF pairs which bind the same DNA sequence (such as YLR131C (Ace2) – YDR146C (Swi5), which implies some antagonistic interaction); (iv) TF pairs with a regulatory (e.g. inhibitory) activity on each other, (such as YPL049C (Dig1) – YHR084W (Ste12)); (v) TF pairs involved in the same biological process (such as YPR104C (Fhl1) – YNL216W (Rap1), both involved in rRNA processing, or YDR146C (Swi5) – YIR018W (Yap5), putatively involved in drug metabolism [20]). Cooperativity between TF pairs without documented relation (neither at protein level nor at functional level) has been occasionally accepted on the basis of cross-talk between different cellular processes, for instance the pair YDR259C (Yap6) – YKL043W (Phd1) might be controlling cell adhesion [20]. Consequently, differences in predictions among the four methods might be the product of the application of different criteria to define cooperativity. Furthermore, some TF pairs considered as false positives by one method are considered *bona fide *cooperative TF pairs in other, for instance YNL216W (Rap1) – YIR018W (Yap5), considered as a potential false positive pair by method C (due to lack of experimental support) and accepted by method N as a part of the same cooperative module.

**Table 2:** Dependence and overlap between the four literature sources.

	**Method N**	**Method B**	**Method T**	**Method C**
**Method N**	**45**	0.0110	0.0061	0.0117
**Method B**	0.206	**31**	0.0068	0.0122
**Method T**	0.132	0.179	**15**	0.0099
**Method C**	0.197	0.222	0.196	**46**

Upper right side: dependence in terms of mutual information between pairs of methods (none of the values was found to be significantly larger than random expectation). Lower left side: overlap in terms of Jaccard coefficient between pairs of methods (all values were found to be significantly larger than random expectation). Diagonal (in bold): number of CTFPs predicted by each method.

When comparing the predictions of different methods, it is also worth mentioning that, although three of the methods derive their information mainly from cell-cycle-related expression analysis, predictions of method N (which is not cell-cycle based) does not show neither a particularly lower dependence nor a lower similarity with the predictions of the other three methods. Although there is a possibility that cooperativity is mainly confined to the control of the cell cycle, we cannot discard a bias towards characterizing cooperative TF pairs involved in the regulation of cell cycle due to (i) the extensive literature available on cell cycle regulation and (ii) the comparison to other prediction methods which are cell-cycle-based.

### Cooperative TF pairs in the protein interaction network

Previous observations suggest an underlying basis of protein-protein interaction for transcriptional cooperativity, either between both TFs or through a non-DNA-binding protein, although other mechanisms not based on protein-protein interactions are possible [1,21]. If one assumes that CTFPs tend to physically interact (either directly or through another protein, which might not bind DNA), the shortest path length between them (i.e. the shortest distance between two cooperative TFs in the PIN) should be shorter than random expectation.

The CTFPs predicted by the four literature methods were not found to be statistically different from one another in terms of their shortest path length in the PIN (Kruskal-Wallis test), which implies some topological consistency across the whole prediction space. When compared to random expectation, the shortest path lengths between members of a CTFP were significantly lower than those produced by random pairing of TFs in all cases (Table 3). This suggests a fast and efficient response through CTFPs, because one member of the CTFP can readily influence the other. This was expected given the necessarily coordinated implication of both members of a cooperative pair in transcriptional control. However, the fraction of directly connected CTFPs are only 40.5%s in the case of method N, 26.9% in the case of method B, 26.7% in the case of method T, and 20.5% in the case of method C. Hence, it seems unlikely that direct physical interaction as a necessary mediator for cooperativity as it is currently defined, highlighting the importance of proteins mediating in this kind of interactions. Interestingly, Table 3 also implies that the fact that two TFs regulate a large number of common target genes (i.e. they are co-regulatory, see *Methods *for details) does not necessarily mean a closeness in the PIN similar to that of CTFPs. Also, all methods predict CTFPs that are significantly closer in the PIN than co-functional TF pairs (co-functional TF pairs are TF pairs which regulate similar cellular functions, see *Methods for details*). This is noteworthy since three methods included in our analysis (all except method N) are largely based in the analysis of the expression patterns of the TFs during the cell cycle, which is known to carry a functional signal [22]. Also, it should be taken into account that it is not at all uncommon for TFs to regulate the transcription of other TFs [23], which results in many of them having similar functional profiles according to our method of establishing co-functionality. Our data, however, seems to suggest that cooperativity determined through the regulatory control of the same biological function(s) does not necessarily imply a cooperative interaction between TFs. However, no significant difference was found for any of the four predicted sets of CTFPs with respect to the set of TF pairs defined by the intersection of co-regulatory and co-functional TF pairs. In other words, TF pairs which are simultaneously co-regulatory and co-functional (hereinafter called co-regulatory ∩ co-functional) show a consistently similar closeness in the PIN (and, consequently, a similar capability of transmitting a signal) to that of the four sets of predicted CTFPs, despite many of them not being defined as cooperative (of all the TF pairs which are co-regulatory ∩ co-functional, 4.76% are predicted as cooperative by method N, 2.38% are predicted as cooperative by method B and none is predicted as cooperative by methods T and C). We have to note, though, that the definition of protein function is inherently incomplete and flawed and, in our case, the function assigned to a TF also depends largely on the quality association between a TF and its target genes. Similar observations were made in the case of the mean shortest path length among the members of a cooperative TF triads [see Additional File 1].

**Table 3:** Shortest path length in the PIN.

	***CTFPs***	***Co-functional TF pairs***		***Co-regulatory TF pairs***		***Co-functional ∩ co-regulatory TF pairs***		***Random TF pairs***	
***Method***	***Mean***	***Mean***	***p-value***	***Mean***	***p-value***	***Mean***	***p-value***	***Mean***	***p-value***

**Method N**	2.119	2.841	1.262·10^-5^	2.967	1.574·10^-5^	1.722	**9.453·10**^-2^	3.151	1.455·10^-10^
**Method B**	2.269	2.841	2.622·10^-3^	2.967	1.534·10^-3^	1.722	**2.347·10**^-2^	3.151	3.229·10^-6^
**Method T**	2.000	2.841	1.757·10^-4^	2.967	3.372·10^-4^	1.722	**1.712·10**^-1^	3.151	7.860·10^-7^
**Method C**	2.256	2.841	2.003·10^-5^	2.967	5.341·10^-5^	1.722	**1.115·10**^-2^	3.151	9.653·10^-10^

Shortest path length between cooperative TF pairs in the PIN. The distribution of shortest path lengths between CTFPs predicted by each method was compared to the distributions in the other sets of TF pairs by means of a Mann-Whitney test. The *p*-value column is in bold type if the distribution of the parameter (in this case, the shortest path length) for a given method is not significantly different to that of the corresponding set (*p*-value < 0.01).

Modularity (i.e. the existence of densely interconnected areas of the network) has been observed in many PINs and has been related to a scale-free architecture of the network [24-27]. TFs in dense modules are expected to show higher topological overlap values (or *modularity *values) in a topological overlap matrix (hereinafter TOM, see *Methods*) [26,28,29]. The CTFPs predicted by the four methods under study were not different from one another in terms of their modularity (Kruskal-Wallis test), which was in all cases higher than expected by random chance (Table 4). Also, the modularity was significantly higher than that observed for co-functional TF pairs in all cases. It was significantly higher than that of co-regulatory TF pairs for the predictions of all methods but method B at *p*-value < 0.01 (but significant at *p*-value < 0.05). Interestingly, however, the modularity was significantly smaller than that observed in TF pairs which were co-regulatory ∩ co-functional for the CTFPs predicted by methods B and C (and method N at *p*-value < 0.05). This adds to the previous observation that there are co-regulatory n co-functional TF pairs that are actually more clustered in the PIN than CTFPs (but are not, however, identified at CTFPs by most of the methods studied). The analysis of the modularity among the members of a cooperative TF triad produced similar results [see Additional File 1]. Results using the noise-filtered version of the PIN and results for CTFPs predicted a different levels of confidence are provided as supplementary information [see Additional File 2 and Additional File 3, respectively].

**Table 4:** Modularity in the PIN.

	***CTFPs***	***Co-functional TF pairs***		***Co-regulatory TF pairs***		***Co-functional ∩ co-regulatory TF pairs***		***Random TF pairs***	
***Method***	***Mean***	***Mean***	***p-value***	***Mean***	***p-value***	***Mean***	***p-value***	***Mean***	***p-value***

**Method N**	0.238	0.071	6.561·10^-6^	0.110	2.048·10^-4^	0.395	**2.054·10**^-2^	0.035	9.321·10^-13^
**Method B**	0.186	0.071	1.582·10^-3^	0.110	**1.020·10**^-2^	0.395	8.690·10^-3^	0.035	2.808·10^-8^
**Method T**	0.212	0.071	1.119·10^-4^	0.110	1.692·10^-3^	0.395	**6.503·10**^-2^	0.035	7.146·10^-9^
**Method C**	0.188	0.071	7.941·10^-8^	0.110	4.160·10^-5^	0.395	4.563·10^-3^	0.035	2.200·10^-16^

Modularity of cooperative TF pairs in the PIN. Modularity was measured as topological overlap (see *Methods*). The distribution of modularity values for the CTFPs predicted by method was compared to distributions in the other sets of TF pairs by means of a Mann-Whitney test. Font styles are as in Table 3.

Modules in the PIN have been related to the function of their members [30-32]. We did not observe correlation between the modularity and the sets of functions regulated by TFs from the whole population of TFs (*ρ *= 0.071, Spearman test; [see Additional file 4]). However, CTFPs exhibited a noticeable correlation (*ρ *= 0.434 for CTFPs predicted by method N, *ρ *= 0.575 for CTFPs predicted by method B, *ρ *= 0.5 for CTFPs predicted by method T, *ρ *= 0.492 for CTFPs predicted by method C, Spearman test), suggesting a tendency for CTFPs to form higher-order cooperative modules controlling the expression of genes with similar function(s).

### Cooperative TF pairs in the regulatory network

The analysis of different aspects of the architecture of the regulatory network can assist in investigating the regulatory association between CTFPs and their target genes, as well as the inter-regulation of CTFPs with other TFs. The regulatory network is a directed graph, which means that a given node (representing a protein in our case) can be connected to other nodes through two types of edges: (i) incoming edges, which denote a regulatory control performed upon the expression of the protein and (ii) outgoing edges, which denote a transcriptional regulatory control performed by the protein (a TF in this case) upon its neighbors.

Being the regulatory network a directed graph, the shortest path length between nodes A and B is measured as the shortest number of edges connecting either node A to node B or node B to node A. In the context of a regulatory network, this measure is similar to that called regulatory closeness [33]. Intuitively, short regulatory path lengths between TFs imply a stronger influence by one TF on the expression of another. The four sets of CTFPs predicted by the four methods under study were not found to be statistically different from one another in terms of their shortest path lengths in this network (Kruskal-Wallis test). Furthermore, predicted CTFPs did not exhibit path lengths significantly shorter than any of the models of TF pairs used for comparison, including the random pairing of TFs (with the only exception in this case of the predictions of method C; Table 5). The lengths of multi-component loop structures (closed regulatory circuits) involving CTFPs were not significantly shorter than expected by random (Mann-Whitney test; mean loop lengths: 7.30, 8.67, 7.38 and 7.27 for CTFPs predicted by the methods N, B, T and C, respectively), which means that cooperativity does not favor small regulatory motifs as an inter-regulatory mechanism of transcription control. Thus, these results suggest that cooperative TFs rarely interact via inter-regulation. Additionally, we did not observe a correlation between the path length in the regulatory network and the co-expression of TF pairs (Spearman test; [see Additional file 5]), which is consistent with previous claims based on the analysis of mRNA expression profiles under a large number of cellular conditions [33]. Interestingly, the mean shortest path length of the cooperative TF triads was significantly shorter than that of the co-functional TF triads and the random TF triads [see Additional File 1]. This leads to the idea that there is a mutual regulation between cooperative TFs at levels of cooperativity higher than cooperative pairs.

**Table 5:** Shortest path length in the regulatory network.

	***CTFPs***	***Co-functional TF pairs***		***Co-regulatory TF pairs***		***Co-functional ∩ co-regulatory TF pairs***		***Random TF pairs***	
***Method***	***Mean***	***Mean***	***p-value***	***Mean***	***p-value***	***Mean***	***p-value***	***Mean***	***p-value***

**Method N**	3.731	3.970	**5.196·10**^-1^	3.292	**4.162·10**^-1^	5.000	**4.779·10**^-1^	4.380	**1.304·10**^-1^
**Method B**	3.500	3.970	**3.498·10**^-1^	3.292	**4.123·10**^-1^	5.000	**5.483·10**^-1^	4.380	**6.550·10**^-2^
**Method T**	2.846	3.970	**5.882·10**^-2^	3.292	**4.878·10**^-1^	5.000	**1.641·10**^-1^	4.380	**1.246·10**^-2^
**Method C**	3.258	3.970	**7.551·10**^-2^	3.292	**9.632·10**^-1^	5.000	**2.659·10**^-1^	4.380	5.292·10^-3^

Shortest path length between cooperative TF pairs in the regulatory network. The distribution of shortest path lengths between the CTFPs predicted by each method was compared to distributions in the other sets of TF pairs by means of a Mann-Whitney test. Font styles are as in Table 3.

Aside from the inter-regulatory associations between TFs, a certain inner community structure has also been observed in the organization of the regulatory network, which can be used to uncover specific roles for CTFPs [34-37]. A TOM was used to measure the extent to which any two TFs shared regulatory partners. Because of the directed nature of the regulatory network, two TOMs were generated: the in-TOM (accounting for incoming edges, which measures the fraction of TFs regulating the expression of any two TFs) and the out-TOM (accounting for outgoing edges, which measures the fraction of genes regulated by of any two TFs). The CTFPs were not found to be statistically different from one another neither in their in-TOM nor in their out-TOM (Kruskal-Wallis test). As shown in Table 6, The in-degree modularity did not show significant differences with random expectation. This observation, together with the results of the analysis of the shortest path length in the same network, reveal that CTFPs are not necessarily co-regulated (i.e. both members of a CTFP tend to integrate unrelated regulatory inputs). The same conclusion can be extracted from the observation of the modularity among members of a predicted cooperative TF triad [see Additional File 1]. The analysis of the out-degree modularity, however, showed that the two members of a CTFP are likely to have a significantly larger number of common target genes than expected by chance (Table 7). The out-degree modularity is not significantly larger than that of co-regulatory TF pairs. Although this could be intuitively expected, it is noteworthy since the prediction of cooperativity by all four methods under study involved the analysis of the *n *target genes common to two TFs (as opposed to the target genes regulated solely by one of them), which may only represent a small fraction of the total number of target genes of both TFs combined (despite the strength of the combinatorial effect of the cooperative TF pairs on the *n *common target genes). Method T explicitly selected TF pairs sharing a significantly large *n*. Its independence-test criterion for assessing significance in this aspect was less strict than ours (and, according to the authors, could be skipped in order to find more potential CTFPs). We also observed in Table 7 that the out-degree modularity was significantly larger for predicted CTFPs with respect to co-functional TF pairs. This result indicates that both members of a CTFP co-regulate the expression of a group of target genes to a larger extent that a co-functional TF pair does. This is not trivial, since the methods studied did not explicitly seek TF pairs whose target genes (common to both TFs or not) displayed similar function(s). Instead, the set of *n *target genes common to both TFs in a CTFP may be involved in the same cellular process, but the set of target genes specific to each TF may contribute to a variety of other processes. The CTFPs did not, however, show a larger modularity than TF pairs which were co-regulatory ∩ co-functional. Taken together, these results show a consistently similar role for all four predictions of CTFPs in the context of the regulatory network, which is only different from random expectation in the case of the out-degree modularity. Analysis of the out-degree modularity for cooperative TF triads gave similar results, although in this case the modularity was also larger than that of TF triads with are co-regulatory n co-functional [see Additional File 1]. Results using CTFPs predicted a different levels of confidence are supplied as supplementary information [see Additional File 3].

**Table 6:** In-degree modularity in the regulatory network.

	***CTFPs***	***Co-functional TF pairs***		***Co-regulatory TF pairs***		***Co-functional ∩ co-regulatory TF pairs***		***Random TF pairs***	
***Method***	***Mean***	***Mean***	***p-value***	***Mean***	***p-value***	***Mean***	***p-value***	***Mean***	***p-value***

**Method N**	0.026	0.057	**3.016·10**^-1^	0.100	**1.176·10**^-1^	0.125	**1.575·10**^-1^	0.044	**4.071·10**^-1^
**Method B**	0.084	0.057	**4.596·10**^-1^	0.100	**8.790·10**^-1^	0.125	**7.560·10**^-1^	0.044	**2.721·10**^-1^
**Method T**	0.083	0.057	**6.551·10**^-1^	0.100	**8.475·10**^-1^	0.125	**7.720·10**^-1^	0.044	**5.095·10**^-1^
**Method C**	0.108	0.057	**9.826·10**^-2^	0.100	**6.728·10**^-1^	0.125	**9.800·10**^-1^	0.044	**2.951·10**^-2^

In-degree modularity of cooperative TF pairs in the regulatory network. The in-degree of a gene denotes the regulatory control performed upon the expression of that gene. Modularity was measured as topological overlap (see *Methods*). The distribution of modularity values for the CTFPs predicted by each method was compared to distributions in the other sets of TF pairs by means of a Mann-Whitney test. Font styles are as in Table 3.

**Table 7:** Out-degree modularity in the regulatory network

	***CTFPs***	***Co-functional TF pairs***		***Co-regulatory TF pairs***		***Co-functional ∩ co-regulatory TF pairs***		***Random TF pairs***	
***Method***	***Mean***	***Mean***	***p-value***	***Mean***	***p-value***	***Mean***	***p-value***	***Mean***	***p-value***

**Method N**	0.424	0.132	1.986·10^-11^	0.318	**1.496·10**^-1^	0.590	1.992·10^-3^	0.050	2.200·10^-16^
**Method B**	0.314	0.132	8.341·10^-7^	0.318	**7.463·10**^-1^	0.590	1.170·10^-4^	0.050	3.553·10^-15^
**Method T**	0.300	0.132	6.995·10^-5^	0.318	**8.042·10**^-1^	0.590	1.210·10^-3^	0.050	5.072·10^-9^
**Method C**	0.314	0.132	3.756·10^-12^	0.318	**8.524·10**^-1^	0.590	5.027·10^-5^	0.050	2.200·10^-16^

Out-degree modularity of cooperative TF pairs in the regulatory network. The out-degree of a gene denotes the regulatory control performed by that gene upon the expression of other genes. Modularity was measured as topological overlap (see *Methods*). The distribution of modularity values for the CTFPs predicted by each method was compared to distributions in the other sets of TF pairs by means of a Mann-Whitney test. Font styles are as in Table 3.

In-degree modularity and out-degree modularity were not correlated, neither in the general population of TFs nor in the case of CTFPs (*ρ *= -0.004 for all TFs, *ρ *= -0.095 for CTFPs, Spearman test [see Additional file 6]. This indicates that CTFPs regulating a certain group of genes are not necessarily co-regulated themselves, therefore supporting cooperativity as mediating in the combination of diverse signals received from more generic regulators.

Finally, modules in the PIN have been related to co-regulation of their members [30,38]. Although one would intuitively expect co-regulation for TFs belonging to the same module, no correlation was observed between the TOM derived from the PIN and in-TOM, meaning that co-regulated TFs are not necessarily more modular (*ρ *= 0.035 for all TFs; *ρ *= -0.057 for CTFPs; Spearman test; [see Additional file 7]). This result agrees with the previously-observed lack of correlation between path length and co-expression and can be partly explained by the role of non-transcriptional regulation of TFs. Notwithstanding direct transcriptional regulation in the presence of promoter-bound TFs [39-41], it is known that many TFs remain at a constitutively low level of expression (sometimes bound to the promoters of their target genes in an inactive state) and their activity is modulated by phosphorylation, cofactors and other post-transcriptional mechanisms [42-45]. Furthermore, different expression levels of a TF may have similar regulatory effects on its target genes. However, a slight positive correlation was found between the modularity in the PIN and the out-TOM for the general population of TFs (*ρ *= 0.137, *p*-value < 10^-5^; Spearman test; [see Additional file 8]). This correlation was clearly stronger if only CTFPs were considered (*ρ *= 0.502, *p*-value < 10^-5^; Spearman test; [see Additional file 5]), which adds to the important role of physical interaction in cooperativity-influenced differential gene expression profiles.

This study highlights the topological commonalities between CTFPs predicted by different methods. Because of that, our observations can be also used to improve current (and future) prediction methods by incorporating topological information. Although not in the scope of this paper, we propose as additional information a simple example of how to integrate our results to score present predictions [see Additional File 9].

## Conclusion

Because prediction of cooperative TFs is critically important for understanding the operation of the regulatory network, our motivation for carrying out this study was to determine whether four different computational methods devised for prediction of CTFPs do detect TF pairs which actually share some consistent features. This is important in the absence of a gold-standard which could be used to benchmark the performance of methods for prediction of transcriptional cooperativity.

The predictions made by the methods under study exhibited low overlap and dependence in their predictions when compared to each other. The PIN-related topological features of the CTFPs detected by the different methods did not vary significantly among them. However, the topological role of the CTFPs in the PIN suggested that cooperativity is indeed reflected in the network as having (i) a shorter path length and (ii) a larger topological overlap than expected by mere chance. This implies a fast access from one member of a CTFP to the other and a tendency to share common interaction partners despite the fact that many CTFPs are not known to directly interact. Also, the topological parameters in the PIN were not significantly distinct to that of TF pairs which are co-regulatory n co-functional, suggesting that, in topological terms, CTFPs behave like those TF pairs despite the fact that many co-regulatory and co-functional TF pairs are not considered CTFPs. From the perspective of the regulatory network, CTFPs were not more inter-regulated than can be explained by chance alone. This observation is consistent across the predictions of all the four sets but one. With no exceptions, the regulatory distance between CTFPs was similar to that of co-functional and co-regulatory TF pairs. Finally, the analysis of the modularity of TF pairs in the regulatory network revealed a consistent lack of a shared regulation for CTFPs, which might result in a role as integrators of varied inputs.

We can conclude from our observations that the predictions drawn from different rationales are consistent with respect to their topological features in networks of different nature such as the protein interaction network and the regulatory network. This suggests that the different predictions analyzed are complementary despite the unclear definition of transcriptional cooperativity. Furthermore, our observations can be used for improving the present prediction methods for characterization of cooperative TFs and for devising new ones, an instrumental task towards unraveling the architecture of transcriptional networks.

## Methods

### Datasets

Cooperative TF pairs (CTFPs) predicted by the four methods were extracted from the literature. The four methods were called method *N, B, C *and *T *[[20,46-48], respectively]. Details on each literature source are available in Table 1. The total number of distinct CTFPs was 91. 14 cooperative groups of three TFs (cooperative TF triads) predicted by method N were also extracted. The authors of the different methods also provided sets of predictions at levels of confidence different than those used in this paper. The analysis of these other predictions is provided [see Additional File 3]. The list of CTFPs and cooperative TF triads in each set is also provided [see Additional file 10]. After excluding TFs which were not considered as such by all methods and transforming all gene names to YPD nomenclature, the resulting dataset contained 101 distinct TFs. Cell-cycle-based expression profiles of the TFs were extracted from Spellman et al. [49].

### Similarities and dependences between the predictions

Pairwise dependences between the CTFPs predicted by the four methods under study were calculated in terms of their mutual information coefficient. The mutual information between the predictions of methods A and B was defined as MI(A, B) = H(A)+H(B)-H(A, B), where H(A) = -Σp(a)·log_2_p(a), H(A, B) = -ΣΣp(a, b)·log_2_p(a, b) and p(a) and p(b) are the marginal probability distributions of the predictions of methods A and B (i.e. the fraction of positive and negative CTFPs identified by each method, respectively). P(a, b) is the joint probability distribution of the predictions of methods A and B. The overlap between the four sets of predictions was calculated by means of the Jaccard coefficient of similarity [50]. The Jaccard coefficient between the predictions of methods A and B is measured as J(A, B) = p(pos, pos)/(1-p(neg, neg)), i.e. the fraction of CTFPs predicted by either method that are predicted by both. The significance of mutual information and Jaccard coefficient for the comparison of two sets of CTFPs was tested against 1000 pairs of random sets of TF of the sizes of the two compared sets.

### Regulatory network and protein interaction network

Associations between TFs and target genes were extracted from Beyer *et al*., who used a Bayesian approach in order to integrate diverse sources with experimental evidences to improve the prediction of this association [51]. We used the subset of TF-regulated gene associations labeled as *highly confident *by the authors. The regulatory network was built as a graph where TFs and regulated genes were represented as nodes and the directed edges represented the control of a TF on the expression target gene. Self-regulatory interactions were excluded. The regulatory network consisted in 3695 proteins and 9959 interactions.

For building a protein interaction network (PIN), we selected all proteins either known to be present in the nucleus or related to transcription (FunCat category 70.10 for nuclear proteins, FunCat category 11.02.03 for transcription-related proteins) [52]. Functional assignments derived from purely computational means were not considered. Proteins were represented as nodes and were connected by an edge if there was evidence of physical interaction between them in the IntAct, MINT, BIND or DIP databases [[53-56], respectively]. PIANA package was used for constructing the network [57]. The resulting PIN consisted of 1900 proteins and 39262 interactions. Because interaction data is known to be noisy, we also generated a filtered PIN composed of interaction supported by more than one independent experimental methods. The results obtained by using this PIN are supplied as additional files [see Additional File 2].

### Topological analysis of the networks

In an undirected network, the shortest path length between two nodes was measured as the smallest number of edges connecting them. In the regulatory network, the shortest path length between two nodes *i *and *j *was calculated as the smallest number of edges connecting either *i *to *j *or *j *to *i*. Lengths of the loops in the regulatory network between two TFs *i *and *j *were calculated as the sum of the shortest distances from *i *to *j *and from *j *to *i*. The *Networkx *module in Python was used for these computations [58].

A topological overlap matrix (TOM) is a matrix which reflects the similarity between each possible pair of nodes in the network in terms of their connectivity (a measure also known as *modularity*). For each pair of nodes *i*, *j *in an undirected network, we define the topological overlap O(*i, j*) as:

$$O_{ij}=\frac{l_{ij}}{\min(k_{i},k_{j})}$$

where *l*_*ij *_denotes the number of common neighbors of *i *and *j *(plus 1 if there is an edge between *i *and *j*) and [min(*k*_*i*_,*k*_*j*_)] is the smaller of the k_*i *_and k_*j *_degrees [26]. In the case of a directed network (such as the regulatory network), the number of common neighbors is calculated independently for incoming edges and outgoing edges. Hence, in the PIN, a topological overlap (or modularity) O_*ij *_= 1 implies that TFs *i *and *j *interact with the same proteins, while O_*ij *_= 0 indicates that *i *and *j *do not share interaction partners. In the regulatory network, O_*ij *_= 1 for the incoming edges implies that both TFs are regulated by the same TFs while O_*ij *_= 1 for the outgoing edges means that both TFs regulate the expression of the same genes.

### Co-functional TF pairs and co-regulatory TF pairs

We wished to obtain a list of TF pairs which regulate the expression of genes with similar functions (referred to as *co-functional TF pairs*). The function of a TF A was defined as a non-binary functional profile $\vec{A}$ of *F *entries, where *F *corresponds to the number different functions considered (*F *= 59 for the second-level categories in the FunCat classification). We placed in the *f*th position the fraction of genes regulated by A which had functions corresponding to the *f*th position. Of the 4248 genes regulated by at least one TF, 3267 were present in at least one second-level functional category. We discarded those TFs regulating genes without functional annotation.

For any pair of TFs A and B in a given dataset, we defined the functional similarity score FS(A, B) as:

$$FS(\vec{A},\vec{B})=\frac{\sum_{i=0}^{f}{\vec{A}}_{i}\cdot {\vec{B}}_{i}}{\sqrt{\sum_{i=0}^{f}\left({\vec{A}}_{i}\cdot {\vec{A}}_{i}\right)\cdot \sum_{i=0}^{f}\left({\vec{B}}_{i}\cdot {\vec{B}}_{i}\right)}}$$

**Figure 1:**
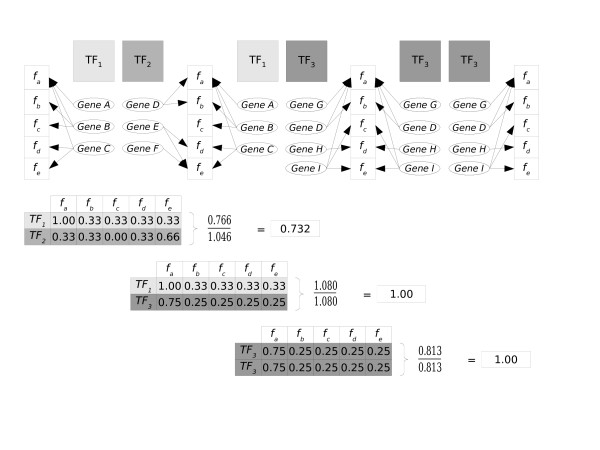
Examples of the calculation of the functional similarity score. Transcription factors are represented as TF_1_, TF_2 _and TF_3_. The group of genes regulated by each TF are G_TF1 _= {A, B, C}, G_TF2 _= {D, E, F} and G_TF3 _= {G, D, H, I}. The five different protein functions in this simplified figure are labeled as *f*_*a*_...*f*_*e*_. The functions are associated to the genes with an arrow. In this example, we calculated the functional similarity score of TF_1_-TF_2_, TF_1_-TF_3 _and TF_3_-TF_3_. The last two examples show how the FS score deals with similar functional profiles.

For any pair of TFs, the FS score ranged from 0 (TFs A and B regulate genes with no function(s) in common) to 1 (TFs A and B regulate genes with exactly the same set of functions). Examples of the calculation of the FS score can be found at Figure 1. We considered two TFs as *co-functional *if their FS score was larger than the 90^th ^percentile of the distribution of FS scores of 1000 randomly paired TFs. The resulting number of co-functional TF pairs was 543.

Also, we wished to obtain a list of TF pairs which regulate a significant number of common target genes (referred to as *co-regulatory TF pairs*). For any pair of TFs, the co-regulatory score was calculated as the number of target genes common to both TFs divided by the mean number of genes shared by the same pair in 1000 random regulatory networks, following Balaji *et al*. [59]. We labeled two TFs as *co-regulatory *if their co-regulatory score was larger than the 90^th ^percentile of the distribution of co-regulatory scores of 1000 randomly paired TFs. The resulting number of co-regulatory TF pairs was 276.

Finally, we identified the group of TF pairs which were simultaneously co-regulatory and co-functional (called *co-regulatory n co-functional TF pairs*). This group contained 42 TF pairs. The complete list of co-functional TF pairs, co-regulatory TF pairs and *co-regulatory n co-functional TF pairs *are available as additional files [see Additional Files 11, 12 and 13].

### Statistical significance

A distribution of 1000 randomly paired TFs was used as a random model to obtain the statistical significance (at a *p*-value < 0.01) of the topological parameters of the network versus its random expectation (using the non-parametric Man-Whitney test). Also, the distribution of the topological parameters of CTFPs predicted by each method was statistically compared to that of: (i) the co-functional TF pairs, (ii) the co-regulatory TF pairs and (iii) the TF pairs which were co-regulatory ∩ co-functional. All calculations in this paper were performed with the R statistical package [60].

## Abbreviations

CTFP, cooperative transcription factor pair; PIN, protein interaction network; TF, transcription factor; TOM, topological overlap matrix.

## Authors' contributions

DA conceived the study and carried out the analysis. BO participated in the design of the study and helped to draft the manuscript. Both authors read and approved the final manuscript.

## Supplementary Material

Additional file 1 Results for the analysis of cooperative TF triads. This file contains the results of the analysis of the members of cooperative TF triads in the framework of the PIN and the regulatory network. Click here for file

Additional file 2 Results for the analysis of the filtered PIN. This file contains the results of the topological analysis of the CTFPs in a PIN created by the accumulation of independent experimental evidence. Because of this, this PIN is deemed to be more reliable. Click here for file

Additional file 3 Results for the analysis of predictions at different levels of confidence. This file contains the results of the topological analysis of CTFPs predicted at levels of confidence different than those used in the main text. Click here for file

Additional file 4 Correlation between functional similarity and modularity in the PIN. Correlation between functional similarity and modularity in the PIN (measured as topological overlap, see the *Methods *section in the paper). Blue dots represent values derived from all TFs. Orange dots represent values derived from CTFPs only. Correlation was calculated by means of a Spearman test. Correlations for each set of CTFPs are as follows: *ρ *= 0.434 (*p*-value = 0.003) for CTFPs predicted by method N, *ρ *= 0.575 (*p*-value = 0.001) for CTFPs predicted by method B, *ρ *= 0.5 (*p*-value = 0.058) for CTFPs predicted by method T, *ρ *= 0.492 (*p*-value = 0.002) for CTFPs predicted by method C. Click here for file

Additional file 5 Correlation between the path length in the regulatory network and the co-expression of TF pairs. Correlation between the path length in the regulatory network and the co-expression of TF pairs. Co-expression was calculated by means of the Pearson correlation coefficient of cell-cycle-based expression data (see *Methods*). Blue dots represent values derived from all TFs. Orange dots represent values derived from CTFPs only. Correlation was calculated by means of a Spearman test. Correlations for each set of CTFPs are as follows: *ρ *= -0.059 (*p*-value = 0.775) for CTFPs predicted by method N, *ρ *= -0.319 (*p*-value = 0.148) for CTFPs predicted by method B, *ρ *= 0.391 (*p*-value = 0.186) for CTFPs predicted by method T, *ρ *= -0.019 (*p*-value = 0.918) for CTFPs predicted by method C. Click here for file

Additional file 6 Correlation between in-degree modularity and out-degree modularity. Correlation between in-degree modularity and out-degree modularity (measured as topological overlap, see the *Methods *section in the paper). Blue dots represent values derived from all TFs. Orange dots represent values derived from CTFPs only. Correlation was calculated by means of a Spearman test. Correlations for each set of CTFPs are as follows: *ρ *= -0.113 (*p*-value = 0.459) for CTFPs predicted by method N, *ρ *= -0.173 (*p*-value = 0.351) for CTFPs predicted by method B, *ρ *= -0.061 (*p*-value = 0.830) for CTFPs predicted by method T, *ρ *= -0.15 (*p*-value = 0.320) for CTFPs predicted by method C. Click here for file

Additional file 7 Correlation between in-degree modularity and modularity in the PIN. Correlation between in-degree modularity and modularity in the PIN (measured as topological overlap, see the *Methods *section in the paper). Blue dots represent values derived from all TFs. Orange dots represent values derived from CTFPs only. Correlation was calculated by means of a Spearman test. Correlations for each set of CTFPs are as follows: *ρ *= 0.178 (*p*-value = 0.243) for CTFPs predicted by method N, *ρ *= -0.207 (*p*-value = 0.265) for CTFPs predicted by method B, *ρ *= -0.138 (*p*-value = 0.625) for CTFPs predicted by method T, *ρ *= -0.151 (*p*-value = 0.318) for CTFPs predicted by method C. Click here for file

Additional file 8 Correlation between out-degree modularity and modularity in the PIN. Correlation between out-degree modularity and modularity in the PIN (measured as topological overlap, see the *Methods *section in the paper). Blue dots represent values derived from all TFs. Orange dots represent values derived from CTFPs only. Correlation was calculated by means of a Spearman test. Correlations for each set of CTFPs are as follows: *ρ *= 0.592 (*p*-value = 2·10^-5^) for CTFPs predicted by method N, *ρ *= 0.727 (*p*-value = 0) for CTFPs predicted by method B, *ρ *= 0.68 (*p*-value = 0.005) for CTFPs predicted by method T, *ρ *= 0.43 (*p*-value = 0.003) for CTFPs predicted by method C. Click here for file

Additional file 9 Example of the use of topological data to score existing predictions of CTFPs. The file contains an example of the used of the observations from our study to score existing predictions of CTFPs. Click here for file

Additional file 10 List of CTFPs predicted by each method. The file contains a list of the CTFPs predicted by each method in two formats: YPD and gene name. Click here for file

Additional file 11 List of co-functional TF pairs. The file contains a tabulated list of the co-functional TF pairs used in our study. Click here for file

Additional file 12 List of co-regulatory TF pairs. The file contains a tabulated list of the co-regulatory TF pairs used in our study. Click here for file

Additional file 13 List of co-functional and co-regulatory TF pairs. The file contains a tabulated list of the co-regulatory n co-functional TF pairs used in our study. Click here for file
