# Supplementary material for: Topological comparison of methods for predicting transcriptional cooperativity in yeast
Source: BMC Genomics. 2008 Mar 25;9:137. doi: 10.1186/1471-2164-9-137 (PMC2315657; doi:10.1186/1471-2164-9-137)
Supplement: Additional file 1 — Results for the analysis of cooperative TF triads. This file contains the results of the analysis of the members of cooperative TF triads in the framework of the PIN and the regulatory network. [file 1471-2164-9-137-S1.pdf]

Additional file #1 for the paper ***Topological comparison of methods for predicting transcriptional cooperativity in yeast*** *by Aguilar & Oliva*.

ANALYSIS OF COOPERATIVE TF TRIADS

**Shortest path length in the PIN**	***CTFTs***	***Co-functional TF triads***		***Co-regulatory TF triads***		***Co-functional ∩ co-regulatory TF triads***		***Random TF triads***	
	Mean	***Mean***	***p-value***	***Mean***	***p-value***	***Mean***	***p-value***	***Mean***	***p-value***
**Method N**	2.212	3.000	$7.334 \cdot 10^{-3}$	3.092	$1.523 \cdot 10^{-3}$	-	-	3.159	$2.876 \cdot 10^{-4}$

Table A1.1. Shortest path length between cooperative TF triads (CTFTs) in the PIN. The shortest path length between the members of a CTFT was calculated as the average distance between the three possible pairs of TFs in the triad. The distribution of shortest path lengths between CTFTs predicted by each method was compared to the distributions in the other sets of TF triads by means of a Mann-Whitney test. The *p*-value column is shaded if the shortest path length distribution for a given method is not significantly different to that of the corresponding set (*p*-value < 0.01). The co-functional ∩ co-regulatory TF triads column is empty because no groups of three TFs could be made satisfying conditions of being simultaneously co-regulatory and co-functional.

**Modularity in the PIN**	***CTFTs***	***Co-functional TF triads***		***Co-regulatory TF triads***		***Co-functional ∩ co-regulatory TF triads***		***Random TF triads***	
	Mean	***Mean***	***p-value***	***Mean***	***p-value***	***Mean***	***p-value***	***Mean***	***p-value***
**Method N**	0.109	0.002	$1.769 \cdot 10^{-4}$	0.000	$3.899 \cdot 10^{-5}$	-	-	0.001	$2.200 \cdot 10^{-16}$

Table A1.2. Modularity of cooperative TF triads in the PIN. Modularity was measured as topological overlap (see *Methods* in the main text). The modularity of the members of a CTFT was calculated as the average modularity of the three possible pairs of TFs in the triad. The distribution of modularity values for the CTFTs predicted by method was compared to distributions in the other sets of TF triads by means of a Mann-Whitney test. The co-functional ∩ co-regulatory TF triads column is empty because no groups of three TFs could be made satisfying conditions of being simultaneously co-regulatory and co-functional. Cell shading is as in Table A1.1.

**Shortest path length in the regulatory network**	***CTFTs***	***Co-functional TF triads***		***Co-regulatory TF triads***		***Co-functional ∩ co-regulatory TF triads***		***Random TF triads***	
	Mean	***Mean***	***p-value***	***Mean***	***p-value***	***Mean***	***p-value***	***Mean***	***p-value***
**Method N**	2.500	4.667	$8.328 \cdot 10^{-3}$	3.524	$1.253 \cdot 10^{-1}$	-	-	4.293	$7.875 \cdot 10^{-3}$

Table A1.3. Shortest path length between cooperative TF triads in the regulatory network. The shortest path length between the members of a CTFT was calculated as the average distance between the three possible pairs of TFs in the triad. The distribution of shortest path lengths between the CTFTs predicted by each method was compared to distributions in the other sets of TF triads by means of a Mann-Whitney test. The co-functional ∩ co-regulatory TF triads column is empty because no groups of three TFs could be made satisfying conditions of being simultaneously co-regulatory and co-functional. Cell shading is as in Table A1.1.

**Modularity in the regulatory network (incoming edges)**	***CTFTs***	***Co-functional TF triads***		***Co-regulatory TF triads***		***Co-functional ∩ co-regulatory TF triads***		***Random TF triads***	
	Mean	***Mean***	***p-value***	***Mean***	***p-value***	***Mean***	***p-value***	***Mean***	***p-value***
**Method N**	0.000	0.000	1	0.000	1	-	-	0.003	$6.280 \cdot 10^{-1}$

Table A1.4. In-degree modularity of cooperative TF triads in the regulatory network. The in-degree of a gene denotes the regulatory control performed upon the expression of that gene. Modularity was measured as topological overlap (see *Methods* in the main text). The modularity of the members of a CTFT was calculated as the average modularity of the three possible pairs of TFs in the triad. The distribution of modularity values for the CTFTs predicted by each method was compared to distributions in the other sets of TF triads by means of a Mann-Whitney test. The co-functional ∩ co-regulatory TF triads column is empty because no groups of three TFs could be made satisfying conditions of being simultaneously co-regulatory and co-functional. Cell shading is as in Table A1.1.

**Modularity in the regulatory network (outgoing edges)**	***CTFTs***	***Co-functional TF triads***		***Co-regulatory TF triads***		***Co-functional ∩ co-regulatory TF triads***		***Random TF triads***	
	Mean	***Mean***	***p-value***	***Mean***	***p-value***	***Mean***	***p-value***	***Mean***	***p-value***
**Method N**	0.311	0.008	$2.200 \cdot 10^{-16}$	0.014	$2.200 \cdot 10^{-16}$	-	-	0.004	$2.200 \cdot 10^{-16}$

Table A1.4. Out-degree modularity of cooperative TF triads in the regulatory network. The out-degree of a gene denotes the regulatory control performed by that gene upon the expression of other genes. Modularity was measured as topological overlap (see *Methods* in the main text). The modularity of the members of a CTFT was calculated as the average modularity of the three possible pairs of TFs in the triad. The distribution of modularity values for the CTFTs predicted by each method was compared to distributions in the other sets of TF triads by means of a Mann-Whitney test. The co-functional ∩ co-regulatory TF triads column is empty because no groups of three TFs could be made satisfying conditions of being simultaneously co-regulatory and co-functional. Cell shading is as in Table A1.1.
