# Supplementary material for: Topological comparison of methods for predicting transcriptional cooperativity in yeast
Source: BMC Genomics. 2008 Mar 25;9:137. doi: 10.1186/1471-2164-9-137 (PMC2315657; doi:10.1186/1471-2164-9-137)
Supplement: Additional file 2 — Results for the analysis of the filtered PIN. This file contains the results of the topological analysis of the CTFPs in a PIN created by the accumulation of independent experimental evidence. Because of this, this PIN is deemed to be more reliable. [file 1471-2164-9-137-S2.pdf]

Additional file #2 for the paper ***Topological comparison of methods for predicting transcriptional cooperativity in yeast*** *by Aguilar & Oliva*.

COMPARISON OF THE PREDICTIONS USING THE FILTERED PROTEIN INTERACTION NETWORK

INTRODUCTION

The reduction of noise in protein interaction networks (PINs) by considering interactions suported by more than one independent experimental evidences has been widely studied and applied (among others, *Pereira-Leal et al., 2004; Lu et al., 2005; Güldener et al., 2006; Xia et al., 2006; von Mering et al., 2007*). We built a new PIN (hereinafter called *filtered PIN*) where the interaction between two proteins was confirmed by more than one experiment. Then, we repeated the topological analysis presented in the main text using this new PIN. Results showed no remarkable differences.

METHODS

For building the filtered PIN, we selected all proteins either known to be present in the nucleus or related to transcription (FunCat category 70.10 for nuclear proteins, FunCat category 11.02.03 for transcription-related proteins) [ref. 52 of the main text]. Functional assignments derived from purely computational means were not considered. Proteins were represented as nodes and were connected by an edge if there was more than one experimental evidence of physical interaction between them in the IntAct, MINT, BIND or DIP databases [refs. 53- 56 of the main text]. PIANA package was used for constructing the network [ref. 57 of the main text]. The filtered PIN consisted of 1830 proteins and 18092 interactions (96.3% of the proteins and 46.1% of the interactions present in the PIN).

RESULTS AND DISCUSSION

Results between the PIN and the filtered PIN were very similar in terms of distance between CTFPs (**Table A2.1**, compare with Table 3 in the main text). Results were also similar in terms of modularity (**Table A2.2**, compare with Table 4 in the main text). The only differences were: i) the modularity of the CTFPs predicted by method T was significantly higher than that of co-regulatory TF pairs at p-value<0.01 (but not at p-value<0.05) and ii) the modularity of the CTFPs predicted by method C and method B was not significantly lower

than of co-regulatory ∩ co-functional TF pairs (in agreement with CTFPs predicted by the other methods).

Because there were not large differences between the analysis carried out with the complete PIN and the analysis carried out with the filtered PIN, we can conclude that the noise present in interaction data does not have a remarkable impact on our study. It has been shown that evidence integration produces a more reliable PIN,which might account for part of the differences observed. However, it is also true that the integration process trades off sensitivity and specificity. Furthermore, it reduces the order of the network as the threshold in the number of concurrent evidences increases. In our case, this reduces the sample size of TF pairs upon which the analysis can be performed, which affects the power of the statistical tests as well as potentially introducing some bias. This reduction of the coverage should also be taken into account when interpreting the observations. Also, in this context, it has to be noted that any filtered PIN is a sample of the original PIN. Consequently, the sampling process will have affected the topological parameters with respect to the original PIN. The reduction in the number of nodes, and more importantly, in the number of edges, will break up the giant component of the network into smaller components. Hence, the contribution of these components to the reduction of the average shortest path length in the PIN will be lost. This would result in an increase of the average shortest path length of the filtered PIN, caused by the loss of information derived from the filtering process.

REFERENCES

Güldener U, Münsterkötter M, Oesterheld M, Pagel P, Ruepp A, Mewes HW, Stümpflen V. **MPact: the MIPS protein interaction resource on yeast**. Nucleic Acids Res. 2006 Jan 1;34(Database issue):D436-41

Lu LJ, Xia Y, Paccanaro A, Yu H, Gerstein M. **Assessing the limits of genomic data integration for predicting protein networks**. Genome Res. 2005 Jul;15(7):945-53

Pereira-Leal JB, Enright AJ, Ouzounis CA. **Detection of functional modules from protein interaction networks**. Proteins. 2004 Jan 1;54(1):49-57

von Mering C, Jensen LJ, Kuhn M, Chaffron S, Doerks T, Krüger B, Snel B, Bork P. **STRING 7--recent developments in the integration and prediction of protein interactions**. Nucleic Acids Res. 2007 Jan; 35(Database issue):D358-62

Xia K, Dong D, Han JD. **IntNetDB v1.0: an integrated protein-protein interaction network database**

generated by a probabilistic model. BMC Bioinformatics. 2006 Nov 18;7:508

Shortest path length in the PIN	*CTFPs*	*Co-functional TF pairs*		*Co-regulatory TF pairs*		*Co-functional ∩ co-regulatory TF pairs*		*Random TF pairs*	
	Mean	*Mean*	*p-value*	*Mean*	*p-value*	*Mean*	*p-value*	*Mean*	*p-value*
**Method N**	2.216	3.486	$7.187 \cdot 10^{-8}$	3.320	$3.682 \cdot 10^{-4}$	1.938	$2.127 \cdot 10^{-1}$	3.926	$3.340 \cdot 10^{-13}$
**Method B**	2.238		$1.916 \cdot 10^{-5}$		$4.938 \cdot 10^{-3}$		$1.596 \cdot 10^{-1}$		$1.069 \cdot 10^{-8}$
**Method T**	2.467		$2.355 \cdot 10^{-3}$		* $5.473 \cdot 10^{-2}$		$1.108 \cdot 10^{-1}$		$3.359 \cdot 10^{-5}$
**Method C**	2.000		$8.964 \cdot 10^{-11}$		$4.681 \cdot 10^{-5}$		$1.580 \cdot 10^{-1}$		$2.200 \cdot 10^{-16}$

Table A2.1. Shortest path length between cooperative TF pairs in the filtered PIN. The distribution of shortest path lengths between CTFPs predicted by each method was compared to the distributions in the other sets of TF pairs by means of a Mann-Whitney test. The *p*-value column is shaded if the shortest path length distribution for a given method is not significantly different to that of the corresponding set (*p*-value < 0.01). An asterisk indicates a result different than that observed with the complete PIN.

**Modularity in the PIN**	***CTFPs***	***Co-functional TF pairs***		***Co-regulatory TF pairs***		***Co-functional ∩ co-regulatory TF pairs***		***Random TF pairs***	
	Mean	***Mean***	***p-value***	***Mean***	***p-value***	***Mean***	***p-value***	***Mean***	***p-value***
**Method N**	0.304	0.071	$5.219 \cdot 10^{-8}$	0.166	$6.239 \cdot 10^{-3}$	0.434	$1.301 \cdot 10^{-1}$	0.027	$2.200 \cdot 10^{-16}$
**Method B**	0.269		$3.342 \cdot 10^{-6}$		$1.794 \cdot 10^{-2}$		* $1.410 \cdot 10^{-1}$		$2.220 \cdot 10^{-16}$
**Method T**	0.227		$3.026 \cdot 10^{-5}$		* $3.752 \cdot 10^{-2}$		$9.307 \cdot 10^{-2}$		$4.241 \cdot 10^{-14}$
**Method C**	0.261		$3.829 \cdot 10^{-12}$		$2.500 \cdot 10^{-4}$		* $1.668 \cdot 10^{-1}$		$2.200 \cdot 10^{-16}$

Table A2.2. Modularity of cooperative TF pairs in the filtered PIN. Modularity was measured as topological overlap (see *Methods* in the main text). The distribution of modularity values for the CTFPs predicted by method was compared to distributions in the other sets of TF pairs by means of a Mann-Whitney test. Cell shading is as in Table A2.1. An asterisk indicates a result different than that observed with the complete PIN.
