# Supplementary material for: Topological comparison of methods for predicting transcriptional cooperativity in yeast
Source: BMC Genomics. 2008 Mar 25;9:137. doi: 10.1186/1471-2164-9-137 (PMC2315657; doi:10.1186/1471-2164-9-137)
Supplement: Additional file 3 — Results for the analysis of predictions at different levels of confidence. This file contains the results of the topological analysis of CTFPs predicted at levels of confidence different than those used in the main text. [file 1471-2164-9-137-S3.pdf]

Additional file #3 for the paper ***Topological comparison of methods for predicting transcriptional cooperativity in yeast*** *by Aguilar & Oliva*.

COMPARISON OF THE PREDICTIONS AT DIFFERENT LEVELS OF CONFIDENCE

INTRODUCTION

In this Additional File, we carry out the topological comparison of the CTFPs predicted by the four methods under study at levels of confidence different that those used in the main text. Differences with respect to results in the main text were in the limit of significance in most cases.

METHODS

The four sets of predicted CTFPs analyzed in this paper were identified at a level of confidence deemed as appropiated by the authors of each method. These predictions, used in the main text of our paper, will hereinafter be referred to as *main sets*. However, predictions at other levels of confidence (higher or lower, or both) were supplied as supplementary data accompanying the papers under study. Method N provided a more stringent set of predictions by integrating protein location data and protein function data to the predictive conditions. This set contained 39 CTFPs. For short, we will refer to this set as *integrated set N*. Predictions made by method B were dependent in a *p*-value (chosen to be P_B<0.001 in the paper). The authors provided a more stringent list of predictions (at P_B<0.0001) and a less stringent list of predictions (at P_B<0.01). The *low-confidence set B* contained 208 CTFPs and the *high-confidence set B* contained 16 CTFPs. As noted by the authors, the functional nature of the TF pairs included in each set was different. Authors of method T provided as supplementary data a list of doubtful predictions which consisted of 29 CTFPs. This set will be called *doubtful set T*. Finally, authors of method C did not explicitly provide predictions at different levels of confidence. They did, however, classify some of their predictions as false positives on the basis of literature support. Hence, we have removed those false positives from their main set of predictions. This set resulted in a total of 44 CTFPs. Because of its nature, this set will be referred to as *true positive set T*. Overlap between the sets was calculated by means of the Jaccard coefficient (see *Methods* in the main text).

RESULTS AND DISCUSSION

The present results should be interpreted in the light of the uneven criteria used by the different authors to set the confidence of their predictions. This resulted in sets of predictions which had diverse overlaps with the main sets (**Table A3.1**). For instance, the *true positive set T* was not very dissimilar to the main set T (Jaccard coefficient = 0.761), which would result in a similar topological behaviour. On the other hand, the *low-confidence set B* had a very low overlap with the main set B (Jaccard coefficient = 0.097). This would make us expect a more different behaviour. Also, it should be taken into account that predictions with lower confidence do not always include predictions made with higher confidence. For instance, the *low-confidence set B* only contains 19 of the 31 TF pairs predicted in the main set B (~61%). The comparison between the distributions of the sets revealed that only *low-confidence set B* shows significant differences with respect to the main set B (for the shortest path length in the PIN, the modularity in the PIN and the out-degree modularity, **Table A3.2**). This could be expected given the low overlap between both sets. For the remaining sets, no statistical difference was found with respect to their corresponding main sets.

Analysis of the shortest path length of the sets of CTFPs in the protein interaction network (PIN) produced, in general terms, similar results to those observed for the main sets (**Table A3.3**). All predictions showed a significantly shorter distance in the PIN than expected by random chance, regardless their level of confidence. This agrees with the main sets and implies that closeness between CTFPs in the PIN is a strong signal for transcriptional cooperativity. The *integrated set N* showed a larger shortest path length than co-functional ∩ co-regulatory TF pairs, although the difference was not statistically significant. The *low-confidence set B* had a longer average path length than the main set B, which was expected if we assume that it contained more noise in the data. Also, it did not show significant differences in terms of shortest path length with respect to co-functional TF pairs and co-regulatory TF pairs. As noted by the authors of the method, the *low-confidence set B* contains TFs that bind to many promoters, which can account for this observation. The *high-confidence set B* shows an average path length between CTFPs similar to that of the main set B. Although it does not show signficantly shorter distance between the members of the pair than do members of a co-functional or a co-regulatory TF pair at p-value<0.01, however, the distance is significantly shorter distance at p-value<0.05. A possible reason for this variation

in *p*-values might be the small size of the set, 16 CTFPs. This makes the analysis prone to statistical small-size effects to which Mann-Whitney test is particularly sensitive (*Siegel, 1956; Kasuya, 2001*). The *doubtful set T* and the *true positive set C* behaved just like their respective main sets.

All predictions consistently showed a significantly larger modularity in the PIN than expected by random chance, regardless their level of confidence (**Table A3.4**). This agrees with the main sets. Statistical differences with other models (i.e. co-functional TF pairs, co-regulatory TF pairs, and co-functional ∩ co-regulatory TF pairs) disagree in some cases with the observations in the main sets. However, the significance of the differences lie beneath the thresholds of *p*-value<0.05 and *p*-value<0.01 for all sets but *low-confidence set B* and *high-confidence set B*. The *integrated set N* showed a slightly lower modularity in the PIN than the main set N, which was unexpected given the higher reliability attributed to this set. A possible reason is that the *integrated set N* was not composed of CTFPs deemed to be statistically more probable, but of a subset of CTFPs which regulated target genes sharing similar functions and cellular locations. This would enrich the set in co-functional CTFPs (which, as seen in the table, have a lower average modularity). The difference of the modularity of the predicted set with respect to the co-regulatory TF pairs was not significant at *p*-value<0.01 (although it was at *p*-value<0.05). Also, the difference of the modularity of the *integrated set N* with respect to the co-regulatory ∩ co-functional TF pairs was significant whereas the main set N was not (although it was at *p*-value<0.05). *Low-confidence set B* showed a much lower average modularity than the main set, as expected for a more generic set of predictions. This modularity was not different to that observed in co-functional TF pairs. *High-confidence set B* was not statistically different in terms of modularity than any of the models except the random model, although the *p*-values were, again, just above the *p*-value<0.01 threshold used throughout this study. The *doubtful set T* showed a behaviour identical to the main set. The *true positives set C* behaved similarly to the main set C, with a difference with respect to co-regulatory ∩ co-functional TF pairs in the limit of the significance.

The analysis of the shortest distance between CTFPs in the regulatory network agreed in general terms with the main sets, regardless the degree of confidence of the sets (**Table A3.5**). The most remarkable deviation from the main sets is method B, where both sets

analyzed (*high-confidence* and, more surprising, *low-confidence*) showed a significantly lower path length than random expectation. As mentioned before, there is a functional separation between CTFPs in both groups. An enrichment of the *low-confidence set* in inter-regulatory TFs could explain this observation, at least partly.

Analysis of the in-degree modularity in the regulatory network showed no large differences between the different levels of confidence and the original results for any method (**Table A3.6**). The *low-confidence set B* and *doubtful set T* were more modular (i.e. shared more regulatory inputs) than expected by mere chance. Both sets were predicted with low confidence and show the lowest overlap with their respective main sets, so these results reinforce the concept of cooperativity as a system which favours the integration of diverse regulatory inputs. With respect to the out-degree modularity, the same both sets had an average outgoing modularity significantly lower than that of co-regulatory TF pairs (**Table A3.6**). Also, this suggests that those sets are rich in TF pairs which share a low number of target genes.

To sum up, the topological analysis of CTFPs predicted at different degrees of confidence revealed no statistical differences versus the CTFPs in the main sets except for the *low-confidence set B*. Topological analysis versus the different models of TF pairs completely agreed with respect to the main sets in (i) shortest path length in the PIN with respect to random TF pairs, (ii) modularity in the PIN with respect to random TF pairs, (iii) out-degree modularity in the regulatory network with respect to random TF pairs, (iv) in-degree modularity in the regulatory network with respect to all models but random TF pairs. This suggests a strong signal for cooperativity for these parameters. Apart from these results, the different sets of predictions showed different levels of agreement with the of observations in the main text, often in the limit of the threshold for significance.

REFERENCES

Kasuya E. 2001. **Mann-Whitney U-test when variances are unequal**. Anim Behav 61:1247–9

Siegel S.1956. **Nonparametric Statistics.** McGraw-Hill, New York.

TABLES

	Jaccard coef.
**Method N vs Method N (integrated set)**	0.313
**Method B vs Method B (low confidence set)**	0.097
**Method B vs Method B (high confidence set)**	0.382
**Method T vs Method T (doubtful set)**	0.158
**Method C vs Method C (true positive set)**	0.761

Table A3.1. Overlap between the main sets and the sets at different levels of confidence. Overlap was calculated in terms of the Jaccard coefficient (see *Methods* in the main text).

	Shortest path length in the PIN	Modularity in the PIN	Shortest path length in the regulatory network	Modularity in the regulatory network (incoming edges)	Modularity in the regulatory network (outgoing edges)
**Method N vs Method N (integrated set)**	$4.143 \cdot 10^{-1}$	$3.451 \cdot 10^{-1}$	$7.122 \cdot 10^{-1}$	$1.000 \cdot 10^{0}$	$3.349 \cdot 10^{-2}$
**Method B vs Method B (low confidence set)**	$6.997 \cdot 10^{-3}$	$7.707 \cdot 10^{-3}$	$8.365 \cdot 10^{-1}$	$6.752 \cdot 10^{-1}$	$2.072 \cdot 10^{-5}$
**Method B vs Method B (high confidence set)**	$9.887 \cdot 10^{-1}$	$9.316 \cdot 10^{-1}$	$7.026 \cdot 10^{-2}$	$9.268 \cdot 10^{-1}$	$8.175 \cdot 10^{-1}$
**Method T vs Method T (doubtful set)**	$5.282 \cdot 10^{-1}$	$6.914 \cdot 10^{-1}$	$1.980 \cdot 10^{-1}$	$4.894 \cdot 10^{-1}$	$1.439 \cdot 10^{-1}$
**Method C vs Method C (true positive set)**	$7.375 \cdot 10^{-1}$	$7.256 \cdot 10^{-1}$	$4.341 \cdot 10^{-1}$	$9.904 \cdot 10^{-1}$	$3.931 \cdot 10^{-1}$

Table A3.2. The distribution of the different parameters of sets of CTFPs predicted at different confidence levels were compared to the main sets. A Mann-Whitney test was used. The *p*-value column is shaded if the shortest path length distribution for a given method is not significantly different to that of the corresponding set (*p*-value < 0.01).

**Shortest path length in the PIN**	***CTFPs***	***Co-functional TF pairs***		***Co-regulatory TF pairs***		***Co-functional ∩ co-regulatory TF pairs***		***Random TF pairs***	
	Mean	***Mean***	***p-value***	***Mean***	***p-value***	***Mean***	***p-value***	***Mean***	***p-value***
**Method N** **integrated set**	2.297		$9.897\cdot10^{-4}$		$3.887\cdot10^{-4}$		* $9.212\cdot10^{-3}$		$2.661\cdot10^{-8}$
**Method B** **low confidence set**	2.831		* $9.989\cdot10^{-1}$		* $1.361\cdot10^{-1}$		* $9.440\cdot10^{-9}$		$2.089\cdot10^{-5}$
**Method B** **high confidence set**	2.267	2.841	* $3.209\cdot10^{-2}$	2.967	* $2.113\cdot10^{-2}$	1.722	$8.850\cdot10^{-2}$	3.151	$1.025\cdot10^{-3}$
**Method T** **doubtful set**	2.217		$1.264\cdot10^{-3}$		$1.128\cdot10^{-3}$		$3.956\cdot10^{-2}$		$2.437\cdot10^{-6}$
**Method C** **true positive set**	2.200		$7.147\cdot10^{-5}$		$1.710\cdot10^{-4}$		$4.366\cdot10^{-2}$		$1.857\cdot10^{-8}$

Table A3.3. Shortest path length between cooperative TF pairs in the PIN. The distribution of shortest path lengths between CTFPs predicted by each method was compared to the distributions in the other sets of TF pairs by means of a Mann-Whitney test. The *p*-value column is shaded if the shortest path length distribution for a given method is not significantly different to that of the corresponding set (*p*-value < 0.01). An asterisk indicates a result different than that of the main set.

**Modularity in the PIN**	***CTFPs***	***Co-functional TF pairs***		***Co-regulatory TF pairs***		***Co-functional ∩ co-regulatory TF pairs***		***Random TF pairs***	
	Mean	***Mean***	***p-value***	***Mean***	***p-value***	***Mean***	***p-value***	***Mean***	***p-value***
**Method N** **integrated set**	0.180	0.071	$3.985 \cdot 10^{-3}$	0.110	* $1.444 \cdot 10^{-2}$	0.395	* $2.435 \cdot 10^{-3}$	0.035	$1.014 \cdot 10^{-7}$
**Method B** **low confidence set**	0.079		* $5.725 \cdot 10^{-1}$		$7.595 \cdot 10^{-1}$		$2.039 \cdot 10^{-10}$		$6.419 \cdot 10^{-7}$
**Method B** **high confidence set**	0.192		* $1.448 \cdot 10^{-2}$		* $3.929 \cdot 10^{-2}$		$3.490 \cdot 10^{-2}$		$2.067 \cdot 10^{-5}$
**Method T** **doubtful set**	0.223		$3.104 \cdot 10^{-4}$		$2.140 \cdot 10^{-3}$		$4.139 \cdot 10^{-2}$		$5.157 \cdot 10^{-9}$
**Method C** **true positive set**	0.210		$4.448 \cdot 10^{-7}$		$7.480 \cdot 10^{-5}$		* $1.818 \cdot 10^{-2}$		$1.110 \cdot 10^{-15}$

Table A3.4. Modularity of cooperative TF pairs in the PIN. Modularity was measured as topological overlap (see *Methods*). The distribution of modularity values for the CTFPs predicted by method was compared to distributions in the other sets of TF pairs by means of a Mann-Whitney test. Cell shading is as in Table A3.3. An asterisk indicates a result different than that of the main set.

**Shortest path length in the regulatory network**	***CTFPs***	***Co-functional TF pairs***		***Co-regulatory TF pairs***		***Co-functional ∩ co-regulatory TF pairs***		***Random TF pairs***	
	Mean	***Mean***	***p-value***	***Mean***	***p-value***	***Mean***	***p-value***	***Mean***	***p-value***
**Method N** **integrated set**	3.393	3.970	$1.866 \cdot 10^{-1}$	3.292	$4.791 \cdot 10^{-1}$	5.000	$8.954 \cdot 10^{-1}$	4.380	$2.391 \cdot 10^{-2}$
**Method B** **low confidence set**	3.438		$3.597 \cdot 10^{-2}$		$4.212 \cdot 10^{-1}$		$4.518 \cdot 10^{-1}$		* $9.181 \cdot 10^{-5}$
**Method B** **high confidence set**	2.214		* $3.167 \cdot 10^{-3}$		$2.175 \cdot 10^{-1}$		$1.159 \cdot 10^{-1}$		* $2.402 \cdot 10^{-4}$
**Method T** **doubtful set**	3.571		$4.523 \cdot 10^{-1}$		$3.756 \cdot 10^{-1}$		$5.307 \cdot 10^{-1}$		$1.011 \cdot 10^{-1}$
**Method C** **true positive set**	2.810		$1.659 \cdot 10^{-2}$		$4.190 \cdot 10^{-1}$		$1.268 \cdot 10^{-1}$		$1.299 \cdot 10^{-3}$

Table A3.5. Shortest path length between cooperative TF pairs in the regulatory network. The distribution of shortest path lengths between the CTFPs predicted by each method was compared to distributions in the other sets of TF pairs by means of a Mann-Whitney test. Cell shading is as in Table A3.3. An asterisk indicates a result different than that of the main set.

**Modularity in the regulatory network (incoming edges)**	***CTFPs***	***Co-functional TF pairs***		***Co-regulatory TF pairs***		***Co-functional ∩ co-regulatory TF pairs***		***Random TF pairs***	
	Mean	***Mean***	***p-value***	***Mean***	***p-value***	***Mean***	***p-value***	***Mean***	***p-value***
**Method N** **integrated set**	0.018	0.057	$2.888 \cdot 10^{-1}$	0.100	$9.363 \cdot 10^{-2}$	0.125	$1.314 \cdot 10^{-1}$	0.044	$3.711 \cdot 10^{-1}$
**Method B** **low confidence set**	0.105		$1.840 \cdot 10^{-2}$		$7.629 \cdot 10^{-1}$		$8.459 \cdot 10^{-1}$		* $5.349 \cdot 10^{-4}$
**Method B** **high confidence set**	0.125		$5.466 \cdot 10^{-1}$		$9.722 \cdot 10^{-1}$		$8.547 \cdot 10^{-1}$		$4.181 \cdot 10^{-1}$
**Method T** **doubtful set**	0.128		$3.297 \cdot 10^{-2}$		$3.825 \cdot 10^{-1}$		$8.690 \cdot 10^{-1}$		* $7.453 \cdot 10^{-3}$
**Method C** **true positive set**	0.112		$1.159 \cdot 10^{-1}$		$6.426 \cdot 10^{-1}$		$1.000 \cdot 10^{0}$		$4.083 \cdot 10^{-2}$

Table A3.6. In-degree modularity of cooperative TF pairs in the regulatory network. The in-degree of a gene denotes the regulatory control performed upon the expression of that gene. Modularity was measured as topological overlap (see *Methods*). The distribution of modularity values for the CTFPs predicted by each method was compared to distributions in the other sets of TF pairs by means of a Mann-Whitney test. Cell shading is as in Table A3.3. An asterisk indicates a result different than that of the main set.

**Modularity in the regulatory network (outgoing edges)**	***CTFPs***	***Co-functional TF pairs***		***Co-regulatory TF pairs***		***Co-functional ∩ co-regulatory TF pairs***		***Random TF pairs***	
	Mean	***Mean***	***p-value***	***Mean***	***p-value***	***Mean***	***p-value***	***Mean***	***p-value***
**Method N** **integrated set**	0.255		$5.740\cdot10^{-7}$		$2.921\cdot10^{-2}$		$2.400\cdot10^{-7}$		$2.200\cdot10^{-16}$
**Method B** **low confidence set**	0.140		* $8.110\cdot10^{-1}$		* $2.200\cdot10^{-16}$		$7.793\cdot10^{-16}$		$2.200\cdot10^{-16}$
**Method B** **high confidence set**	0.306	0.132	$8.228\cdot10^{-6}$	0.318	$9.328\cdot10^{-1}$	0.590	$1.205\cdot10^{-3}$	0.050	$8.001\cdot10^{-11}$
**Method T** **doubtful set**	0.244		$8.563\cdot10^{-3}$		* $6.382\cdot10^{-3}$		$1.836\cdot10^{-6}$		$5.922\cdot10^{-11}$
**Method C** **true positive set**	0.358		$3.275\cdot10^{-11}$		$2.185\cdot10^{-1}$		$1.587\cdot10^{-3}$		$2.200\cdot10^{-16}$

Table A3.7. Out-degree modularity of cooperative TF pairs in the regulatory network. The out-degree of a gene denotes the regulatory control performed by that gene upon the expression of other genes. Modularity was measured as topological overlap (see *Methods*). The distribution of modularity values for the CTFPs predicted by each method was compared to distributions in the other sets of TF pairs by means of a Mann-Whitney test. Cell shading is as in Table A3.3. An asterisk indicates a result different than that of the main set.
