# Supplementary material for: Topological comparison of methods for predicting transcriptional cooperativity in yeast
Source: BMC Genomics. 2008 Mar 25;9:137. doi: 10.1186/1471-2164-9-137 (PMC2315657; doi:10.1186/1471-2164-9-137)
Supplement: Additional file 5 — Correlation between the path length in the regulatory network and the co-expression of TF pairs. Correlation between the path length in the regulatory network and the co-expression of TF pairs. Co-expression was calculated by means of the Pearson correlation coefficient of cell-cycle-based expression data (see Methods). Blue dots represent values derived from all TFs. Orange dots represent values derived from CTFPs only. Correlation was calculated by means of a Spearman test. Correlations for each set of CTFPs are as follows: ρ = -0.059 (p-value = 0.775) for CTFPs predicted by method N, ρ = -0.319 (p-value = 0.148) for CTFPs predicted by method B, ρ = 0.391 (p-value = 0.186) for CTFPs predicted by method T, ρ = -0.019 (p-value = 0.918) for CTFPs predicted by method C. [file 1471-2164-9-137-S5.pdf]

Additional file #5 for the paper ***Topological comparison of methods for predicting transcriptional cooperativity in yeast*** *by Aguilar & Oliva*.

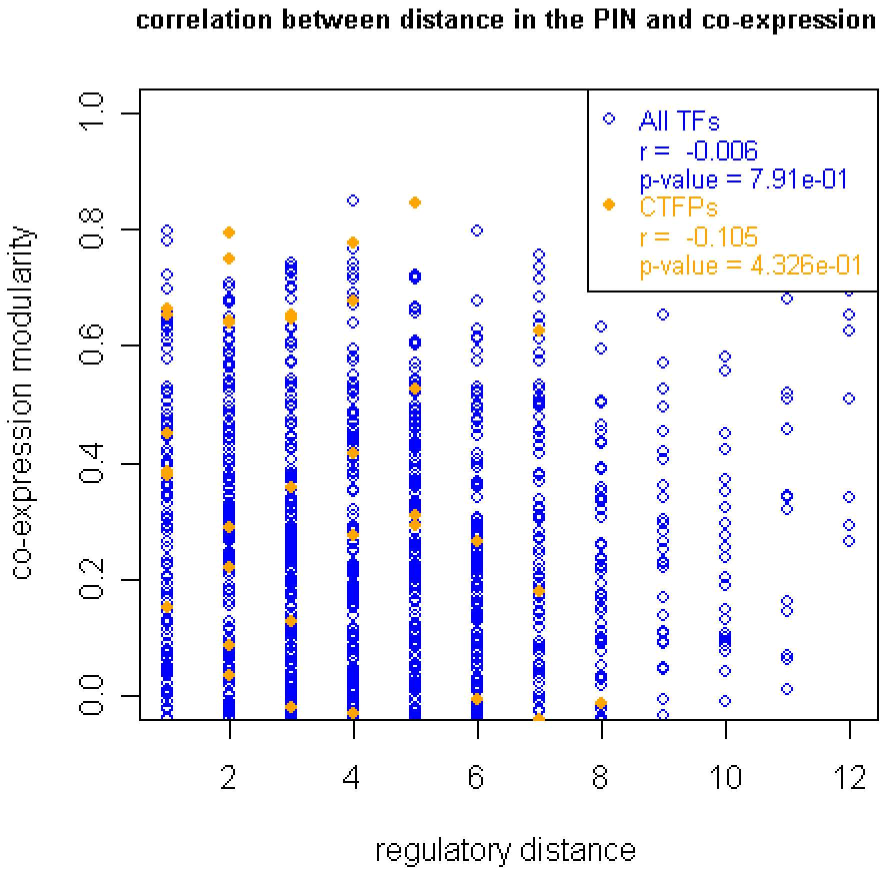

Additional file 5. Correlation between the path length in the regulatory network and the co-expression of TF pairs. Co-expression was calculated by means of the Pearson correlation coefficient of cell-cycle-based expression data (see *Methods*). Blue dots represent values derived from all TFs. Orange dots represent values derived from CTFPs only. Correlation was calculated by means of a Spearman test. Correlations for each set of CTFPs are as follows: ρ = -0.059 (*p*-value = 0.775) for CTFPs predicted by method N, ρ = -0.319 (*p*-value = 0.148) for CTFPs predicted by method B, ρ = 0.391 (*p*-value = 0.186) for CTFPs predicted by method T, ρ = -0.019 (*p*-value = 0.918) for CTFPs predicted by method C.
