# Supplementary material for: Topological comparison of methods for predicting transcriptional cooperativity in yeast
Source: BMC Genomics. 2008 Mar 25;9:137. doi: 10.1186/1471-2164-9-137 (PMC2315657; doi:10.1186/1471-2164-9-137)
Supplement: Additional file 6 — Correlation between in-degree modularity and out-degree modularity. Correlation between in-degree modularity and out-degree modularity (measured as topological overlap, see the Methods section in the paper). Blue dots represent values derived from all TFs. Orange dots represent values derived from CTFPs only. Correlation was calculated by means of a Spearman test. Correlations for each set of CTFPs are as follows: ρ = -0.113 (p-value = 0.459) for CTFPs predicted by method N, ρ = -0.173 (p-value = 0.351) for CTFPs predicted by method B, ρ = -0.061 (p-value = 0.830) for CTFPs predicted by method T, ρ = -0.15 (p-value = 0.320) for CTFPs predicted by method C. [file 1471-2164-9-137-S6.pdf]

Additional file #6 for the paper ***Topological comparison of methods for predicting transcriptional cooperativity in yeast*** *by Aguilar & Oliva*.

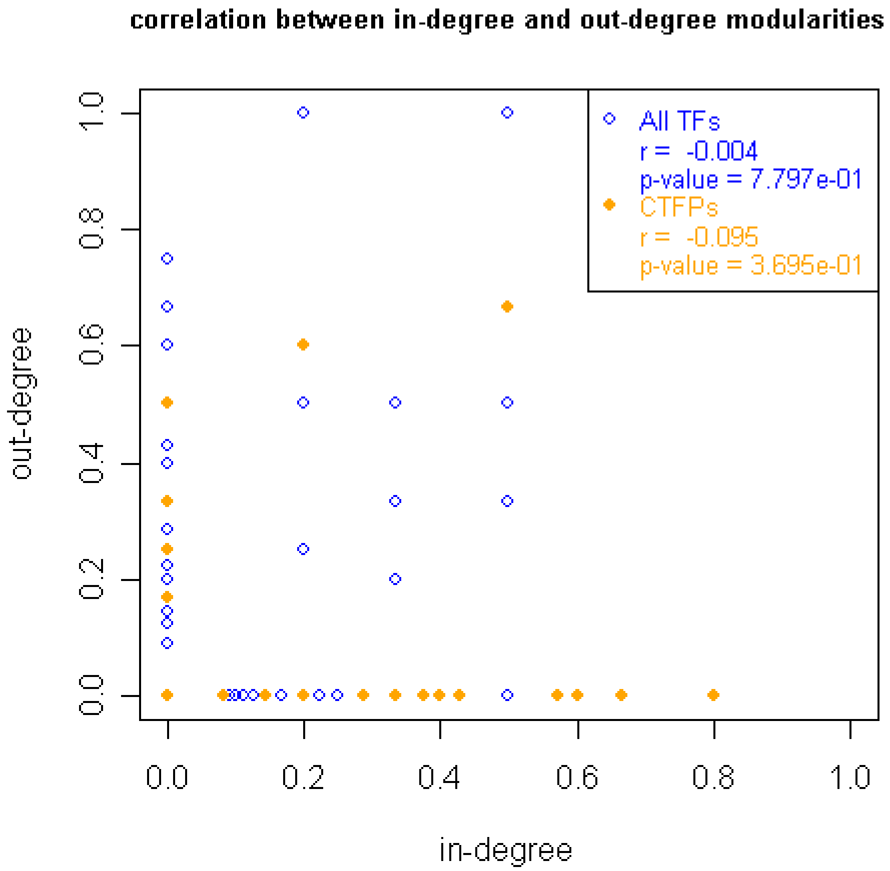

Additional file 6. Correlation between in-degree modularity and out-degree modularity (measured as topological overlap, see the *Methods* section in the paper). Blue dots represent values derived from all TFs. Orange dots represent values derived from CTFPs only. Correlation was calculated by means of a Spearman test. Correlations for each set of CTFPs are as follows: ρ = -0.113 (*p*-value = 0.459) for CTFPs predicted by method N, ρ = -0.173 (*p*-value = 0.351) for CTFPs predicted by method B, ρ = -0.061 (*p*-value = 0.830) for CTFPs predicted by method T, ρ = -0.15 (*p*-value = 0.320) for CTFPs predicted by method C.
