# Supplementary material for: Topological comparison of methods for predicting transcriptional cooperativity in yeast
Source: BMC Genomics. 2008 Mar 25;9:137. doi: 10.1186/1471-2164-9-137 (PMC2315657; doi:10.1186/1471-2164-9-137)
Supplement: Additional file 9 — Example of the use of topological data to score existing predictions of CTFPs. The file contains an example of the used of the observations from our study to score existing predictions of CTFPs. [file 1471-2164-9-137-S9.pdf]

Additional file #9 for the paper ***Topological comparison of methods for predicting transcriptional cooperativity in yeast*** *by Aguilar & Oliva.*

COMBINING TOPOLOGICAL DATA TO SCORE PREDICTED COOPERATIVE TRANSCRIPTION FACTOR PAIRS

INTRODUCTION

Combination of different evidences has been used as a means to improve the accuracy of the prediction of different characteristics of genes and networks *(von Mering et al., 2002; Troyanskaya et al., 2003; von Mering et al., 2003; Karaoz et al., 2004; Zhang et al., 2004)*. A number of different approaches have been implemented to this end, ranging from a simple voting function to Bayesian classifiers and neural networks, always aiming to keep a good trade-off between sensitivity and specificity. The results presented in the main text could be used to improve the performance of existing methods by priorizing those predicted cooperative TF pairs (CTFPs) which comply with certain topological rules. In this additional file, we propose an approach to illustrate the use of our results to improve current predictions by giving a degree of reliability to their results. Since this study is based in the CTFPs predicted by four different methods, we scored the predictions of each method by applying the topological information derived from the analysis of the remaining three. The resulting scored list agrees well with the number of evidences supporting each predicted CTFP.

METHODS

In our study, we measured the topological characteristics of CTFPs predicted by four methods in the frame of two distinct biological networks: protein interaction network (PIN) and regulatory network (RN). CTFPs predicted by each method were compared to four different models of TF pairs: co-functional, co-regulatory, co-regulatory ∩ co-functional and random TF pairs. In order to score the predictions made by each method, we derived from the results a set of topological rules which were common to the remaining three methods. This way, we simulated the improvement of integrating our data on the predictions of each method. For each CTFP predicted by the method being tested, we calculated its *p*-value from the accumulative distribution of each model and each parameter. The unsigned logarithm of the *p*-values will be accumulated to the score of the pair. **Figures A9.1** and **A9.2** illustrate the process of calculation of the score for a CTFP for a particular case. This scoring scheme was

used to incorporate the evidences where all methods agreed. Certain *p*-values could not be calculated for some CTFPs because of the lack of information (e.g. one of the members of the pair was not present in the current protein interaction databases). In those cases, the corresponding *p*-value could not be added to the score. Because we were using an accumulative distribution function to calculate the *p*-values, we set a limit of 10^{-5} for those cases where the resulting *p*-value=0. All the models except the random TF pairs are not mutually independent. Although complex methods exist in order to estimate the loss in the significancy of the contributions for a number of mutually dependent models (*Bailey & Grundy, 1999*), we chosed a conservative approach to correct for the independency assumption by assuming that the dependent models were exactly identical (i.e. completely dependent). In this extreme case, the combination of their *p*-values would simply be 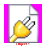 where *p* is the product of their *p*-values and n = 3 (there are three dependent models). Naturally, our data lies between this case and complete independence, but we preferred underestimating the contributions of the dependent models in order to err on the cautious side. A set of 1000 random TF pairs was used to assess the *p*-value of observing the same score (or lower) by mere chance. Correlation between the score of a CTFP and the number of evidences supporting it was calculated by means of the Pearson correlation coefficient.

RESULTS AND DISCUSSION

To evaluate the quality of any prediction method, we need to measure the relevance of the predictions against a gold-standard. As explained in the main text, an experimentally verified gold-standard is lacking in the case of transcriptionally cooperative TF pairs. For this reason, authors in related literature rely on different kinds of experimental evidences to evaluate the quality of their predictions, which produces ambiguous results. If TFs A and B are predicted to cooperate, but no prior biological knowledge links them in this aspect of their functionality, is that wrong prediction, or a relevant biological discovery? We decided to use the number of concurrent predictions as a reasonable reflection of the current knowledge on cooperativity between TFs, despite the problems that this naive scheme may have. For this reason, also, this evaluation is conservative: we do not predict new CTFPs but only give a new support to

previously-made predictions.

The scores assigned to all CTFPs are shown in **Tables 1a** to **1e**. Not all CTFPs predicted by each method are present in the table since no not all TFs are present in the PIN or in the RN. For all methods except method N, we found a significant positive correlation between the score of a TF pair and the number of other methods which predicted its cooperativity (ρ=0.194, *p*-value=$2.019 \cdot 10^{-1}$ for CTFPs predicted by method N, ρ=0.795, *p*-value=$9.03 \cdot 10^{-8}$ for CTFPs predicted by method B, ρ=0.796, *p*-value=$3.781 \cdot 10^{-4}$ for CTFPs predicted by method T and ρ=0.763, *p*-value=$6.6651 \cdot 10^{-10}$ for CTFPs predicted by method C). The reason for the low correlation between scores and number of evidences for method N could be explained, at least partly, by the fact that it is the only method not explicitly limited to cell-cycle-related cooperativity. It is interesting to note that the some of the highest-scoring CTFP are detected only by one method. For instance, the cooperation between YDL106C (Pho2) and YFR034C (Pho4) was only detected by method N (see main text for reference). Being both TFs critically important in response to phosphate starvation (Barbaric *et al.*, 1998), they are reasonably good candidates for cooperativity. Similarly, the TF pair YPL075W (Gcr1) and YNL199C (Gcr2) is also ranked among the top positions despite being detected by method N only. Well-known CTFPs such as YNL068C (Fkh2) – YMR043W (Mcm1) and YNL068C (Fkh2) – YOR372C (Ndd1) are validated a with high scores as well.

Finally, we would like stress that we presented here a very simple example of the use of our results to help improving prediction of CTFPs. More complex approaches are possible (and even desirable) in order to integrate topological data into existing and future methods for prediction of transcriptional cooperativity.

REFERENCES

Bailey TL, Grundy WN. **Classifying proteins by family using the product of correlated p-value**s. Third international conference on computational molecular biology (RECOMB99), pp. 10-14, Association for Computing Machinery, New York, April, 1999.

Barbaric S, Münsterkötter M, Goding C, Hörz W. **Cooperative Pho2-Pho4 interactions at the PHO5 promoter are critical for binding of Pho4 to UASp1 and for efficient transactivation by Pho4 at UASp2**. Mol Cell Biol. 1998 May;18(5):2629-39

Karaoz U, Murali TM, Letovsky S, Zheng Y, Ding C, Cantor CR, Kasif S. **Whole-genome annotation by using evidence integration in functional-linkage networks**. Proc Natl Acad Sci U S A. 2004 Mar 2;101(9):2888-93

Troyanskaya OG, Dolinski K, Owen AB, Altman RB, Botstein D. **A Bayesian framework for combining heterogeneous data sources for gene function prediction (in *Saccharomyces cerevisiae*)**. Proc Natl Acad Sci U S A. 2003 Jul 8;100(14):8348-53

von Mering C, Krause R, Snel B, Cornell M, Oliver SG, Fields S, Bork P. **Comparative assessment of large-scale data sets of protein-protein interactions**. Nature. 2002 May 23;417(6887):399-403

von Mering C, Huynen M, Jaeggi D, Schmidt S, Bork P, Snel B. **STRING: a database of predicted functional associations between proteins**. Nucleic Acids Res. 2003 Jan 1;31(1):258-61

Zhang LV, Wong SL, King OD, Roth FP. **Predicting co-complexed protein pairs using genomic and proteomic data integration**. BMC Bioinformatics. 2004 Apr 16;5:38

Additional file #9 for the paper ***Topological comparison of methods for predicting transcriptional cooperativity in yeast*** *by Aguilar & Oliva.*

FIGURES

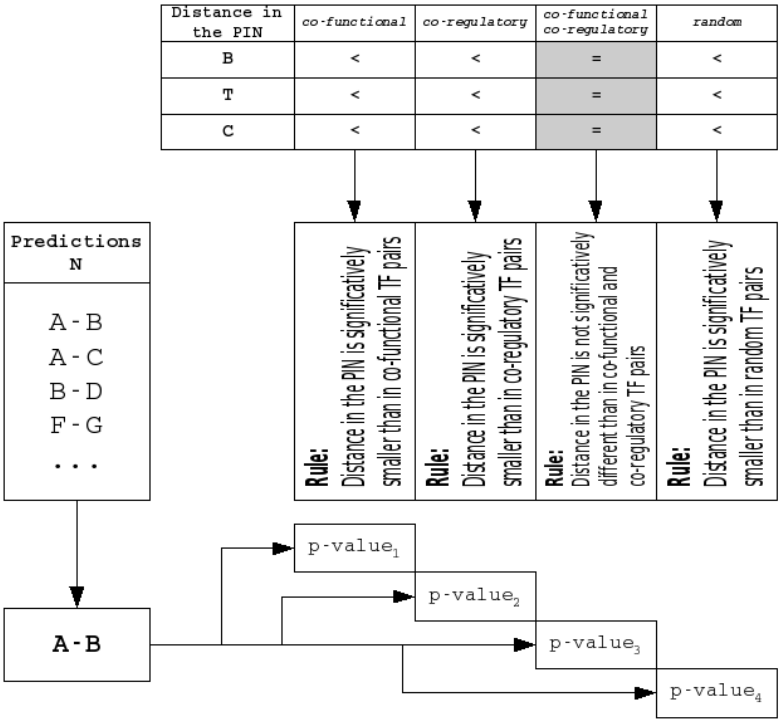

$$\mathbf{score}^{dist^{PIN}}_{A,B} = (|\log(p\text{-}value1)|+|\log(p\text{-}value2)|+|\log(p\text{-}value3)|/3)+|\log(p\text{-}value4)| \qquad 1$$

Figure A9.1. Process of calculation of the score for a $CTFP_{AB}$ predicted by method N considering the information of the shortest path length in the PIN. We wish to score a $CTFP_{AB}$ predicted by method N, so we use only data obtained from the comparison of methods B, T and C. Since all three methods agree in their statistical comparison with the four models (namely, *i)* co-functional model, *ii)* co-regulatory model, *iii)* co-functional ∩ co-regulatory model, *iv)* random model), all four pieces of evidence can be used. The agreement between the methods is shown by the fact that all columns show either empty cells (statistically significant difference) or shaded cells (no statistical difference). In this case, CTFPs predicted by all methods have a distance in the PIN significantly shorter than all models but the co-functional ∩ co-regulatory TF pairs. The distance in the PIN between TFs A and B was obtained and the probability that it could be observed in any of the models was measured using an accumulative distribution function. Then, the score for $CTFP_{AB}$ based on the shortest path length in the PIN was calculated as the mean of the unsigned logarithm of the *p*-values of the three first models (in order to minimize the effect of mutual dependences) plus the unsigned logarithm of the *p*-value of the random model. This score would add up to similar scores calculated from other parameters (namely, modularity in the PIN, shortest path length in the RN, in-degree modularity in the RN and out-degree modularity in the RN).

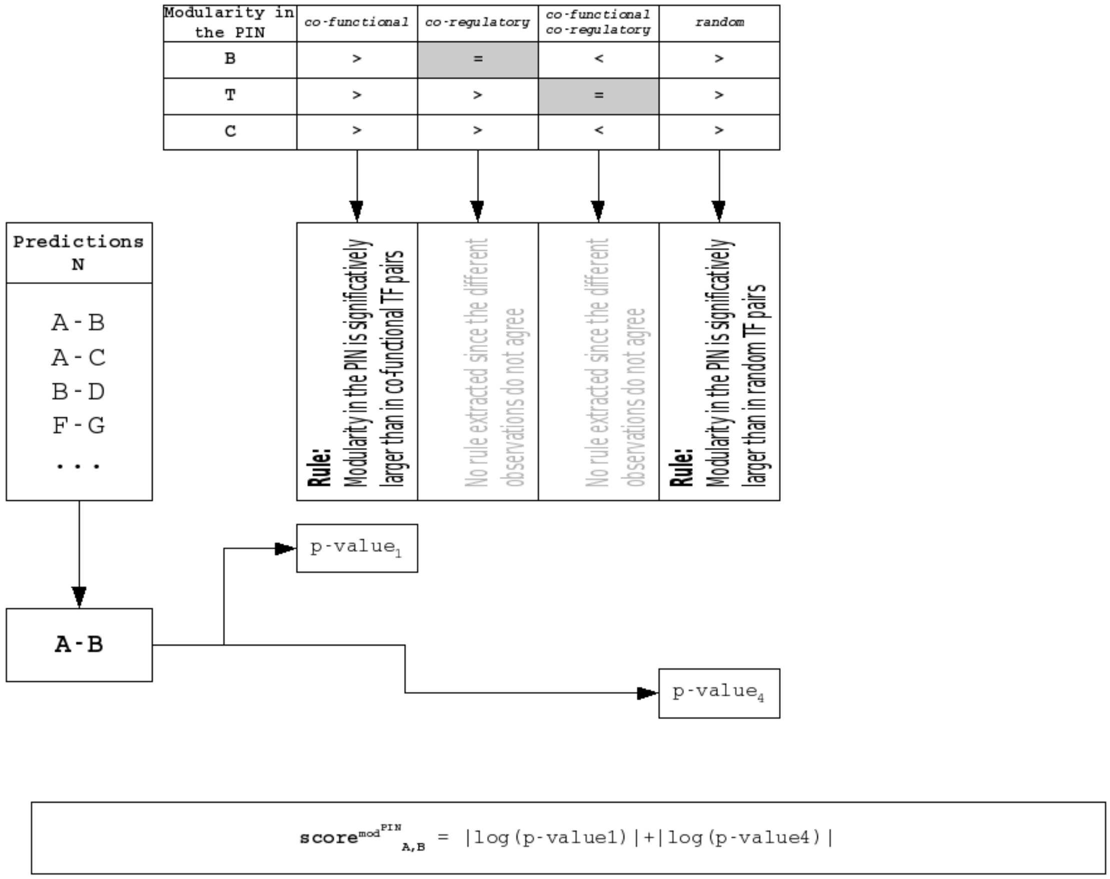

2

Figure A9.2. Process of calculation of the score for a $CTFP_{AB}$ predicted by method N considering the information of the modularity the PIN. We wish to score a $CTFP_{AB}$ predicted by method N, so we use only data obtained from the comparison of methods B, T and C. Not all three methods agree in their statistical comparison with the four models (i.e. *i)* co-functional, *ii)* co-regulatory, *iii)* co-functional ∩ co-regulatory, *iv)* random). Hence, we consider as informative only the two pieces of evidence were all methods agree (i.e. the columns without a mixture of empty and shaded cells). In this case, all methods have a modularity larger than that of co-functional TF pairs and larger than random expectation. The distance in the PIN between TFs A and B was obtained and the probability that it could be observed in any of the models was measured using an accumulative distribution function. Then, the score for $CTFP_{AB}$ based on the shortest path length in the PIN was calculated as the sum of the unsigned logarithm of the *p*-value of the first models and the unsigned logarithm of the *p*-value of the random model. Since both models are independent, no correction is necessary in this case. This score would add up to similar scores calculated from other parameters (namely, shortest path length in the PIN, shortest path length in the RN, in-degree modularity in the RN and out-degree modularity in the RN).

TABLES

TF1 (YPD name)	TF2 (YPD name)	TF1 (gene name)	TF2 (gene name)	# evid	score	*p*-value
YDL106C	YFR034C	PHO2	PHO4	0	17.150	7.843137·10-4
YNL216W	YIR018W	RAP1	YAP5	1	16.581	1.568627·10-3
YGL073W	YNL216W	HSF1	RAP1	0	16.461	2.352941·10-3
YDR146C	YIR018W	SWI5	YAP5	1	16.352	3.921569·10-3
YOR372C	YHR206W	NDD1	SKN7	1	16.352	3.921569·10-3
YHR206W	YML007W	SKN7	YAP1	0	16.213	6.274510·10-3
YKL043W	YDR259C	PHD1	YAP6	0	16.213	6.274510·10-3
YMR043W	YER111C	MCM1	SWI4	0	16.213	6.274510·10-3
YNL068C	YMR043W	FKH2	MCM1	3	15.498	7.058824·10-3
YNL068C	YOR372C	FKH2	NDD1	3	15.444	7.843137·10-3
YIL131C	YNL068C	FKH1	FKH2	2	14.975	1.019608·10-2
YIL131C	YOR372C	FKH1	NDD1	3	14.975	1.019608·10-2
YMR043W	YOR372C	MCM1	NDD1	3	14.975	1.019608·10-2
YPL075W	YNL199C	GCR1	GCR2	0	14.845	1.176471·10-2
YBL008W	YOR038C	HIR1	HIR2	1	14.845	1.176471·10-2
YOR372C	YML007W	NDD1	YAP1	0	14.646	1.254902·10-2
YGL013C	YNL216W	PDR1	RAP1	0	14.048	1.333333·10-2
YDL056W	YMR043W	MBP1	MCM1	0	12.745	1.411765·10-2
YDL056W	YHR206W	MBP1	SKN7	0	12.259	1.490196·10-2
YER111C	YLR182W	SWI4	SWI6	3	12.011	1.647059·10-2
YDL056W	YER111C	MBP1	SWI4	1	12.011	1.647059·10-2
YMR043W	YLR182W	MCM1	SWI6	1	11.862	1.725490·10-2
YPR104C	YIR018W	FHL1	YAP5	0	11.581	1.803922·10-2
YDR043C	YKL043W	NRG1	PHD1	1	11.377	1.882353·10-2
YLR013W	YNL216W	GAT3	RAP1	1	11.258	1.960784·10-2
YNL309W	YER111C	STB1	SWI4	2	9.975	2.039216·10-2
YMR312W	YOR358W	ELP6	HAP5	0	9.845	2.745098·10-2
YBL021C	YOR358W	HAP3	HAP5	0	9.845	2.745098·10-2
YMR042W	YML099C	ARGR1	ARGR2	1	9.845	2.745098·10-2
YHR187W	YMR312W	IKI1	ELP6	0	9.845	2.745098·10-2
YDL056W	YLR182W	MBP1	SWI6	3	9.845	2.745098·10-2
YNL309W	YLR182W	STB1	SWI6	1	9.845	2.745098·10-2

YGL237C	YOR358W	HAP2	HAP5	0	9.845	2.745098·10-2
YGL237C	YBL021C	HAP2	HAP3	0	9.845	2.745098·10-2
YGL237C	YMR312W	HAP2	ELP6	0	9.845	2.745098·10-2
YDL056W	YKL062W	MBP1	MSN4	0	9.725	2.823529·10-2
YDR043C	YDR259C	NRG1	YAP6	1	9.700	2.901961·10-2
YHR187W	YOR358W	IKI1	HAP5	0	9.377	3.058824·10-2
YPR104C	YNL216W	FHL1	RAP1	0	9.377	3.058824·10-2
YHR187W	YBL021C	IKI1	HAP3	0	9.048	3.372549·10-2
YNL216W	YLR403W	RAP1	SFP1	0	9.048	3.372549·10-2
YPR104C	YLR403W	FHL1	SFP1	0	9.048	3.372549·10-2
YPR104C	YGL013C	FHL1	PDR1	0	9.048	3.372549·10-2
YLR013W	YIR018W	GAT3	YAP5	1	8.750	3.450980·10-2
YPR104C	YLR013W	FHL1	GAT3	1	6.258	3.529412·10-2

Table A9a. Scored cooperative TF pairs predicted by method N.

Additional file #9 for the paper ***Topological comparison of methods for predicting transcriptional cooperativity in yeast*** *by Aguilar & Oliva.*

**TF1 (YPD name)**	**TF2 (YPD name)**	**TF1 (gene name)**	**TF2 (gene name)**	**# evid**	**score**	***p*-value**
YNL068C	YMR043W	FKH2	MCM1	3	15.498	7.058824·10-3
YNL068C	YOR372C	FKH2	NDD1	3	15.444	7.843137·10-3
YIL131C	YNL068C	FKH1	FKH2	2	14.975	1.019608·10-2
YIL131C	YOR372C	FKH1	NDD1	3	14.975	1.019608·10-2
YMR043W	YOR372C	MCM1	NDD1	3	14.975	1.019608·10-2
YBL008W	YOR038C	HIR1	HIR2	1	14.845	1.176471·10-2
YER111C	YLR182W	SWI4	SWI6	3	12.011	1.647059·10-2
YDR043C	YKL043W	NRG1	PHD1	1	11.377	1.882353·10-2
YNL309W	YER111C	STB1	SWI4	2	9.975	2.039216·10-2
YMR042W	YML099C	ARGR1	ARGR2	1	9.845	2.745098·10-2
YDL056W	YLR182W	MBP1	SWI6	3	9.845	2.745098·10-2
YDR043C	YDR259C	NRG1	YAP6	1	9.700	2.901961·10-2
YPR104C	YLR013W	FHL1	GAT3	1	6.258	3.529412·10-2
YEL009C	YDR310C	GCN4	SUM1	0	3.995	3.843137·10-2
YLR131C	YDR146C	ACE2	SWI5	1	3.040	4.078431·10-2
YOR028C	YDR259C	CIN5	YAP6	1	2.792	4.549020·10-2
YOR372C	YNL309W	NDD1	STB1	1	2.792	4.549020·10-2
YGL073W	YBR049C	HSF1	REB1	0	2.783	4.627451·10-2
YOR028C	YDR043C	CIN5	NRG1	0	2.602	4.784314·10-2
YBR049C	YHR206W	REB1	SKN7	0	2.602	4.784314·10-2
YBR182C	YDR146C	SMP1	SWI5	0	2.508	4.862745·10-2
YGL073W	YHR206W	HSF1	SKN7	0	1.748	5.098039·10-2
YML099C	YEL009C	ARGR2	GCN4	0	0.950	5.960784·10-2
YLR131C	YGL073W	ACE2	HSF1	0	0.950	5.960784·10-2
YLR131C	YBR049C	ACE2	REB1	0	0.950	5.960784·10-2
YKL062W	YIR018W	MSN4	YAP5	1	0.322	6.352941·10-2
YPL248C	YMR182C	GAL4	RGM1	0	0.298	6.823529·10-2
YIR023W	YDR463W	DAL81	STP1	0	0.298	6.823529·10-2
YGL013C	YBR182C	PDR1	SMP1	1	0.000	1.000000·10^{0}
YLR013W	YGL013C	GAT3	PDR1	1	0.000	1.000000·10^{0}
YLR013W	YKL062W	GAT3	MSN4	1	0.000	1.000000·10^{0}

Table A9b. Scored cooperative TF pairs predicted by method N.

**TF1 (YPD name)**	**TF2 (YPD name)**	**TF1 (gene name)**	**TF2 (gene name)**	**# evid**	**score**	***p*-value**
YOR372C	YHR206W	NDD1	SKN7	1	16.352	3.921569·10-3
YNL068C	YMR043W	FKH2	MCM1	3	15.498	7.058824·10-3
YNL068C	YOR372C	FKH2	NDD1	3	15.444	7.843137·10-3
YIL131C	YOR372C	FKH1	NDD1	3	14.975	1.019608·10-2
YMR043W	YOR372C	MCM1	NDD1	3	14.975	1.019608·10-2
YER111C	YLR182W	SWI4	SWI6	3	12.011	1.647059·10-2
YDL056W	YLR182W	MBP1	SWI6	3	9.845	2.745098·10-2
YNL068C	YLR182W	FKH2	SWI6	1	4.324	3.607843·10-2
YPL089C	YDR146C	RLM1	SWI5	0	3.995	3.843137·10-2
YKR099W	YGL071W	BAS1	AFT1	0	2.931	4.156863·10-2
YOR372C	YER111C	NDD1	SWI4	1	2.792	4.549020·10-2
YOR372C	YNL309W	NDD1	STB1	1	2.792	4.549020·10-2
YNL068C	YER111C	FKH2	SWI4	1	2.463	5.019608·10-2
YIL131C	YMR043W	FKH1	MCM1	1	1.225	5.647059·10-2
YKR099W	YBL008W	BAS1	HIR1	0	0.298	6.823529·10-2

Table A9c. Scored cooperative TF pairs predicted by method T.

Additional file #9 for the paper ***Topological comparison of methods for predicting transcriptional cooperativity in yeast*** *by Aguilar & Oliva.*

**TF1** (YPD name)	**TF2** (YPD name)	**TF1** (gene name)	**TF2** (gene name)	**# evid**	**score**	***p*-value**
YNL216W	YIR018W	RAP1	YAP5	1	16.279	1.568627·10-3
YDR146C	YIR018W	SWI5	YAP5	1	15.976	3.921569·10-3
YNL068C	YMR043W	FKH2	MCM1	3	15.534	7.058824·10-3
YNL068C	YOR372C	FKH2	NDD1	3	15.507	7.843137·10-3
YIL131C	YNL068C	FKH1	FKH2	2	15.038	1.019608·10-2
YIL131C	YOR372C	FKH1	NDD1	3	15.038	1.019608·10-2
YMR043W	YOR372C	MCM1	NDD1	3	15.038	1.019608·10-2
YER111C	YLR182W	SWI4	SWI6	3	11.716	1.647059·10-2
YDL056W	YER111C	MBP1	SWI4	1	11.716	1.647059·10-2
YMR043W	YLR182W	MCM1	SWI6	1	11.471	1.725490·10-2
YLR013W	YNL216W	GAT3	RAP1	1	10.956	1.960784·10-2
YNL309W	YER111C	STB1	SWI4	2	10.038	2.039216·10-2
YDL056W	YLR182W	MBP1	SWI6	3	9.845	2.745098·10-2
YNL309W	YLR182W	STB1	SWI6	1	9.845	2.745098·10-2
YLR013W	YIR018W	GAT3	YAP5	1	8.750	3.450980·10-2
YNL068C	YLR182W	FKH2	SWI6	1	3.671	3.607843·10-2
YNL068C	YDL056W	FKH2	MBP1	0	3.342	3.843137·10-2
YIL131C	YDL056W	FKH1	MBP1	0	3.023	3.921569·10-2
YHR206W	YLR182W	SKN7	SWI6	0	2.655	4.078431·10-2
YLR131C	YDR146C	ACE2	SWI5	1	2.655	4.078431·10-2
YOR028C	YDR259C	CIN5	YAP6	1	2.498	4.470588·10-2
YOR372C	YER111C	NDD1	SWI4	1	2.498	4.470588·10-2
YOR372C	YLR182W	NDD1	SWI6	0	2.498	4.470588·10-2
YKL109W	YIR018W	HAP4	YAP5	0	2.417	4.549020·10-2
YGL013C	YIR018W	PDR1	YAP5	0	2.193	4.941176·10-2
YNL068C	YER111C	FKH2	SWI4	1	2.169	5.019608·10-2
YHR206W	YER111C	SKN7	SWI4	0	1.316	5.411765·10-2
YIL131C	YLR182W	FKH1	SWI6	0	1.316	5.411765·10-2
YPL089C	YER111C	RLM1	SWI4	0	1.316	5.411765·10-2
YPL089C	YLR182W	RLM1	SWI6	0	1.316	5.411765·10-2
YIL131C	YMR043W	FKH1	MCM1	1	1.288	5.568627·10-2
YDL056W	YOR372C	MBP1	NDD1	0	1.288	5.568627·10-2
YMR182C	YIR018W	RGM1	YAP5	0	1.250	5.647059·10-2

YPL049C	YHR084W	DIG1	STE12	0	1.095	5.725490·10-2
YHR084W	YER111C	STE12	SWI4	0	0.959	6.039216·10-2
YLR013W	YKL109W	GAT3	HAP4	0	0.689	6.117647·10-2
YKL109W	YGL013C	HAP4	PDR1	0	0.627	6.274510·10-2
YDL056W	YNL309W	MBP1	STB1	0	0.627	6.274510·10-2
YKL062W	YIR018W	MSN4	YAP5	1	0.322	6.352941·10-2
YHR084W	YLR182W	STE12	SWI6	0	0.298	6.823529·10-2
YGL013C	YMR182C	PDR1	RGM1	0	0.298	6.823529·10-2
YKL062W	YGL013C	MSN4	PDR1	0	0.298	6.823529·10-2
YGL013C	YBR182C	PDR1	SMP1	1	0.000	$1.000000 \cdot 10^{0}$
YLR013W	YGL013C	GAT3	PDR1	1	0.000	$1.000000 \cdot 10^{0}$
YLR013W	YMR182C	GAT3	RGM1	0	0.000	$1.000000 \cdot 10^{0}$
YLR013W	YKL062W	GAT3	MSN4	1	0.000	$1.000000 \cdot 10^{0}$

Table A9d. Scored cooperative TF pairs predicted by method C.
