# Supplementary material for: Topological comparison of methods for predicting transcriptional cooperativity in yeast
Source: BMC Genomics. 2008 Mar 25;9:137. doi: 10.1186/1471-2164-9-137 (PMC2315657; doi:10.1186/1471-2164-9-137)
Supplement: Additional file 10 — List of CTFPs predicted by each method. The file contains a list of the CTFPs predicted by each method in two formats: YPD and gene name. [file 1471-2164-9-137-S10.pdf]

Additional file #10 for the paper ***Topological comparison of methods for predicting transcriptional cooperativity in yeast*** *by Aguilar & Oliva*.

Method	TF1 (YPD code)	TF2 (YPD code)	TF3 (YPD code)	TF1 (gene name)	TF2 (gene name)	TF3 (gene name)
Banerjee & Zhang, 2003	YDR043C	YKL043W		NRG1	PHD1	
Banerjee & Zhang, 2003	YGL073W	YHR206W		HSF1	SKN7	
Banerjee & Zhang, 2003	YLR131C	YGL073W		ACE2	HSF1	
Banerjee & Zhang, 2003	YIR023W	YDR463W		DAL81	STP1	
Banerjee & Zhang, 2003	YOR372C	YNL309W		NDD1	STB1	
Banerjee & Zhang, 2003	YML099C	YEL009C		ARGR2	GCN4	
Banerjee & Zhang, 2003	YNL309W	YER111C		STB1	SWI4	
Banerjee & Zhang, 2003	YOR028C	YDR259C		CIN5	YAP6	
Banerjee & Zhang, 2003	YLR131C	YDR146C		ACE2	SWI5	
Banerjee & Zhang, 2003	YBR182C	YDR146C		SMP1	SWI5	
Banerjee & Zhang, 2003	YOR028C	YDR043C		CIN5	NRG1	
Banerjee & Zhang, 2003	YIL131C	YNL068C		FKH1	FKH2	
Banerjee & Zhang, 2003	YEL009C	YDR310C		GCN4	SUM1	
Banerjee & Zhang, 2003	YPL248C	YMR182C		GAL4	RGM1	
Banerjee & Zhang, 2003	YGL073W	YBR049C		HSF1	REB1	
Banerjee & Zhang, 2003	YDL056W	YLR182W		MBP1	SWI6	
Banerjee & Zhang, 2003	YMR043W	YOR372C		MCM1	NDD1	
Banerjee & Zhang, 2003	YNL068C	YMR043W		FKH2	MCM1	
Banerjee & Zhang, 2003	YNL068C	YOR372C		FKH2	NDD1	
Banerjee & Zhang, 2003	YBL008W	YOR038C		HIR1	HIR2	
Banerjee & Zhang, 2003	YGL013C	YBR182C		PDR1	SMP1	
Banerjee & Zhang, 2003	YER111C	YLR182W		SWI4	SWI6	
Banerjee & Zhang, 2003	YLR013W	YGL013C		GAT3	PDR1	
Banerjee & Zhang, 2003	YPR104C	YLR013W		FHL1	GAT3	
Banerjee & Zhang, 2003	YDR043C	YDR259C		NRG1	YAP6	
Banerjee & Zhang, 2003	YLR013W	YKL062W		GAT3	MSN4	
Banerjee & Zhang, 2003	YBR049C	YHR206W		REB1	SKN7	
Banerjee & Zhang, 2003	YLR131C	YBR049C		ACE2	REB1	
Banerjee & Zhang, 2003	YMR042W	YML099C		ARGR1	ARGR2	
Banerjee & Zhang, 2003	YIL131C	YOR372C		FKH1	NDD1	
Banerjee & Zhang, 2003	YKL062W	YIR018W		MSN4	YAP5	
Nagamine et al., 2005	YMR043W	YLR182W		MCM1	SWI6	
Nagamine et al., 2005	YER111C	YLR182W		SWI4	SWI6	
Nagamine et al., 2005	YDL106C	YFR034C		PHO2	PHO4	
Nagamine et al., 2005	YDL056W	YLR182W		MBP1	SWI6	
Nagamine et al., 2005	YMR043W	YOR372C		MCM1	NDD1	
Nagamine et al., 2005	YNL309W	YER111C		STB1	SWI4	
Nagamine et al., 2005	YNL309W	YLR182W		STB1	SWI6	
Nagamine et al., 2005	YLR013W	YIR018W		GAT3	YAP5	
Nagamine et al., 2005	YKL043W	YDR259C		PHD1	YAP6	
Nagamine et al., 2005	YNL068C	YOR372C		FKH2	NDD1	
Nagamine et al., 2005	YOR372C	YML007W		NDD1	YAP1	
Nagamine et al., 2005	YLR013W	YNL216W		GAT3	RAP1	
Nagamine et al., 2005	YPR104C	YLR403W		FHL1	SFP1	
Nagamine et al., 2005	YDL056W	YER111C		MBP1	SWI4	
Nagamine et al., 2005	YHR187W	YBL021C		IKI1	HAP3	
Nagamine et al., 2005	YHR187W	YMR312W		IKI1	ELP6	
Nagamine et al., 2005	YGL237C	YBL021C		HAP2	HAP3	
Nagamine et al., 2005	YGL237C	YMR312W		HAP2	ELP6	
Nagamine et al., 2005	YGL013C	YNL216W		PDR1	RAP1	
Nagamine et al., 2005	YHR206W	YML007W		SKN7	YAP1	
Nagamine et al., 2005	YPL075W	YNL199C		GCR1	GCR2	
Nagamine et al., 2005	YDL056W	YHR206W		MBP1	SKN7	
Nagamine et al., 2005	YOR372C	YHR206W		NDD1	SKN7	
Nagamine et al., 2005	YDR146C	YIR018W		SWI5	YAP5	
Nagamine et al., 2005	YGL073W	YNL216W		HSF1	RAP1	

Additional file #10 for the paper ***Topological comparison of methods for predicting transcriptional cooperativity in yeast*** *by Aguilar & Oliva*.

Nagamine et al., 2005	YIL131C	YNL068C	FKH1	FKH2
Nagamine et al., 2005	YDL056W	YMR043W	MBP1	MCM1
Nagamine et al., 2005	YDR043C	YDR259C	NRG1	YAP6
Nagamine et al., 2005	YBL021C	YOR358W	HAP3	HAP5
Nagamine et al., 2005	YMR312W	YOR358W	ELP6	HAP5
Nagamine et al., 2005	YHR187W	YOR358W	IKI1	HAP5
Nagamine et al., 2005	YGL237C	YOR358W	HAP2	HAP5
Nagamine et al., 2005	YPR104C	YIR018W	FHL1	YAP5
Nagamine et al., 2005	YPR104C	YLR013W	FHL1	GAT3
Nagamine et al., 2005	YNL216W	YLR403W	RAP1	SFP1
Nagamine et al., 2005	YDR043C	YKL043W	NRG1	PHD1
Nagamine et al., 2005	YMR042W	YML099C	ARGR1	ARGR2
Nagamine et al., 2005	YDL056W	YKL062W	MBP1	MSN4
Nagamine et al., 2005	YNL068C	YMR043W	FKH2	MCM1
Nagamine et al., 2005	YPR104C	YGL013C	FHL1	PDR1
Nagamine et al., 2005	YIL131C	YOR372C	FKH1	NDD1
Nagamine et al., 2005	YNL216W	YIR018W	RAP1	YAP5
Nagamine et al., 2005	YMR043W	YER111C	MCM1	SWI4
Nagamine et al., 2005	YPR104C	YNL216W	FHL1	RAP1
Nagamine et al., 2005	YBL008W	YOR038C	HIR1	HIR2
Tsai et al., 2005	YER111C	YLR182W	SWI4	SWI6
Tsai et al., 2005	YPL089C	YDR146C	RLM1	SWI5
Tsai et al., 2005	YOR372C	YER111C	NDD1	SWI4
Tsai et al., 2005	YOR372C	YNL309W	NDD1	STB1
Tsai et al., 2005	YOR372C	YHR206W	NDD1	SKN7
Tsai et al., 2005	YMR043W	YOR372C	MCM1	NDD1
Tsai et al., 2005	YIL131C	YMR043W	FKH1	MCM1
Tsai et al., 2005	YIL131C	YOR372C	FKH1	NDD1
Tsai et al., 2005	YNL068C	YMR043W	FKH2	MCM1
Tsai et al., 2005	YNL068C	YOR372C	FKH2	NDD1
Tsai et al., 2005	YNL068C	YER111C	FKH2	SWI4
Tsai et al., 2005	YNL068C	YLR182W	FKH2	SWI6
Tsai et al., 2005	YDL056W	YLR182W	MBP1	SWI6
Tsai et al., 2005	YKR099W	YGL071W	BAS1	AFT1
Tsai et al., 2005	YKR099W	YBL008W	BAS1	HIR1
Tsai et al., 2005	YDL056W	YLR182W	MBP1	SWI6
Tsai et al., 2005	YKR099W	YGL071W	BAS1	AFT1
Tsai et al., 2005	YKR099W	YBL008W	BAS1	HIR1
Chang et al., 2006	YDR146C	YIR018W	SWI5	YAP5
Chang et al., 2006	YMR182C	YIR018W	RGM1	YAP5
Chang et al., 2006	YGL013C	YBR182C	PDR1	SMP1
Chang et al., 2006	YKL109W	YGL013C	HAP4	PDR1
Chang et al., 2006	YGL013C	YMR182C	PDR1	RGM1
Chang et al., 2006	YLR013W	YMR182C	GAT3	RGM1
Chang et al., 2006	YHR206W	YER111C	SKN7	SWI4
Chang et al., 2006	YDL056W	YNL309W	MBP1	STB1
Chang et al., 2006	YIL131C	YMR043W	FKH1	MCM1
Chang et al., 2006	YMR043W	YLR182W	MCM1	SWI6
Chang et al., 2006	YPL089C	YLR182W	RLM1	SWI6
Chang et al., 2006	YPL089C	YER111C	RLM1	SWI4
Chang et al., 2006	YHR206W	YLR182W	SKN7	SWI6
Chang et al., 2006	YLR131C	YDR146C	ACE2	SWI5
Chang et al., 2006	YDL056W	YOR372C	MBP1	NDD1
Chang et al., 2006	YPL049C	YHR084W	DIG1	STE12
Chang et al., 2006	YNL216W	YIR018W	RAP1	YAP5
Chang et al., 2006	YKL062W	YGL013C	MSN4	PDR1
Chang et al., 2006	YLR013W	YNL216W	GAT3	RAP1
Chang et al., 2006	YHR084W	YER111C	STE12	SWI4
Chang et al., 2006	YHR084W	YLR182W	STE12	SWI6
Chang et al., 2006	YNL309W	YER111C	STB1	SWI4
Chang et al., 2006	YIL131C	YDL056W	FKH1	MBP1
Chang et al., 2006	YKL109W	YIR018W	HAP4	YAP5
Chang et al., 2006	YLR013W	YKL109W	GAT3	HAP4
Chang et al., 2006	YOR372C	YER111C	NDD1	SWI4

Additional file #10 for the paper ***Topological comparison of methods for predicting transcriptional cooperativity in yeast*** *by Aguilar & Oliva*.

Chang et al., 2006	YOR028C	YDR259C		CIN5	YAP6	
Chang et al., 2006	YNL309W	YLR182W		STB1	SWI6	
Chang et al., 2006	YNL068C	YMR043W		FKH2	MCM1	
Chang et al., 2006	YLR013W	YKL062W		GAT3	MSN4	
Chang et al., 2006	YKL062W	YIR018W		MSN4	YAP5	
Chang et al., 2006	YOR372C	YLR182W		NDD1	SWI6	
Chang et al., 2006	YIL131C	YOR372C		FKH1	NDD1	
Chang et al., 2006	YLR013W	YGL013C		GAT3	PDR1	
Chang et al., 2006	YIL131C	YLR182W		FKH1	SWI6	
Chang et al., 2006	YNL068C	YDL056W		FKH2	MBP1	
Chang et al., 2006	YMR043W	YOR372C		MCM1	NDD1	
Chang et al., 2006	YNL068C	YER111C		FKH2	SWI4	
Chang et al., 2006	YGL013C	YIR018W		PDR1	YAP5	
Chang et al., 2006	YDL056W	YLR182W		MBP1	SWI6	
Chang et al., 2006	YNL068C	YOR372C		FKH2	NDD1	
Chang et al., 2006	YDL056W	YER111C		MBP1	SWI4	
Chang et al., 2006	YLR013W	YIR018W		GAT3	YAP5	
Chang et al., 2006	YNL068C	YLR182W		FKH2	SWI6	
Chang et al., 2006	YIL131C	YNL068C		FKH1	FKH2	
Chang et al., 2006	YER111C	YLR182W		SWI4	SWI6	
Nagamine et al., 2005	YPR104C	YGL013C	YNL216W	FHL1	PDR1	RAP1
Nagamine et al., 2005	YGL237C	YMR312W	YOR358W	HAP2	ELP6	HAP5
Nagamine et al., 2005	YGL237C	YBL021C	YOR358W	HAP2	HAP3	HAP5
Nagamine et al., 2005	YHR187W	YMR312W	YOR358W	IKI1	ELP6	HAP5
Nagamine et al., 2005	YHR187W	YBL021C	YOR358W	IKI1	HAP3	HAP5
Nagamine et al., 2005	YPR104C	YNL216W	YIR018W	FHL1	RAP1	YAP5
Nagamine et al., 2005	YDL056W	YMR043W	YER111C	MBP1	MCM1	SWI4
Nagamine et al., 2005	YPR104C	YNL216W	YLR403W	FHL1	RAP1	SFP1
Nagamine et al., 2005	YPR104C	YLR013W	YNL216W	FHL1	GAT3	RAP1
Nagamine et al., 2005	YOR372C	YHR206W	YML007W	NDD1	SKN7	YAP1
Nagamine et al., 2005	YIL131C	YNL068C	YOR372C	FKH1	FKH2	NDD1
Nagamine et al., 2005	YDR043C	YKL043W	YDR259C	NRG1	MCM1	YAP6
Nagamine et al., 2005	YPR104C	YLR013W	YIR018W	FHL1	GAT3	YAP5

Additional file 10. List of CTFPs and cooperative TF triads predicted by each method.

References are provided in the paper.
